# Immediate impact of yogic breathing on pulsatile cerebrospinal fluid dynamics

**DOI:** 10.1038/s41598-022-15034-8

**Published:** 2022-06-28

**Authors:** Selda Yildiz, John Grinstead, Andrea Hildebrand, John Oshinski, William D. Rooney, Miranda M. Lim, Barry Oken

**Affiliations:** 1 Department of Neurology, Oregon Health & Science University, 3181 SW Sam Jackson Park Road, Portland, OR 97239 USA; 2 Siemens Medical Solutions USA, Inc, Portland, OR 97239 USA; 3 Biostatistics and Design Program, Oregon Health & Science University, Portland, OR 97239 USA; 4 Radiology & Imaging Sciences and Biomedical Engineering, Emory School of Medicine, Emory University, Atlanta, GA 30322 USA; 5 Advanced Imaging Research Center, Oregon Health & Science University, Portland, OR 97239 USA; 6 Department of Behavioral Neuroscience, Oregon Health & Science University, Portland, OR 97239 USA; 7 Knight Cardiovascular Institute, Oregon Health & Science University, Portland, OR 97239 USA; 8 Department of Biomedical Engineering, Oregon Health & Science University, Portland, OR 97239 USA; 9 VA Portland Health Care System, Portland, OR 97239 USA; 10 Division of Pulmonary and Critical Care Medicine, Department of Medicine, Oregon Health & Science University, Portland, OR 97239 USA; 11 Oregon Institute of Occupational Health Sciences, Oregon Health & Science University, Portland, OR 97239 USA

**Keywords:** Imaging, Biomedical engineering, Neuroscience, Magnetic resonance imaging

## Abstract

Cerebrospinal fluid (CSF), a clear fluid bathing the central nervous system (CNS), undergoes pulsatile movements. Together with interstitial fluid, CSF plays a critical role for the removal of waste products from the brain, and maintenance of the CNS health. As such, understanding the mechanisms driving CSF movement is of high scientific and clinical impact. Since pulsatile CSF dynamics is sensitive and synchronous to respiratory movements, we are interested in identifying potential integrative therapies such as yogic breathing to regulate CSF dynamics, which has not been reported before. Here, we investigated the pre-intervention baseline data from our ongoing randomized controlled trial, and examined the impact of four yogic breathing patterns: (i) slow, (ii) deep abdominal, (iii) deep diaphragmatic, and (iv) deep chest breathing with the last three together forming a yogic breathing called three-part breath. We utilized our previously established non-invasive real-time phase contrast magnetic resonance imaging approach using a 3T MRI instrument, computed and tested differences in single voxel CSF velocities (instantaneous, respiratory, cardiac 1st and 2nd harmonics) at the level of foramen magnum during spontaneous versus yogic breathing. In examinations of 18 healthy participants (eight females, ten males; mean age 34.9 ± 14 (SD) years; age range: 18–61 years), we observed immediate increase in cranially-directed velocities of instantaneous-CSF 16–28% and respiratory-CSF 60–118% during four breathing patterns compared to spontaneous breathing, with the greatest changes during deep abdominal breathing (28%, *p* = 0.0008, and 118%, *p* = 0.0001, respectively). Cardiac pulsation was the primary source of pulsatile CSF motion except during deep abdominal breathing, when there was a comparable contribution of respiratory and cardiac 1st harmonic power [0.59 ± 0.78], suggesting respiration can be the primary regulator of CSF depending on the individual differences in breathing techniques. Further work is needed to investigate the impact of sustained training yogic breathing on pulsatile CSF dynamics for CNS health.

## Introduction

### Cerebrospinal fluid

Cerebrospinal fluid (CSF) is one of the two discrete fluid compartments of the brain along with interstitial fluid (ISF), and is crucial for the health of central nervous system (CNS). With the advances in imaging technologies and recent research efforts^1–11^, it is clear that CSF is more than a mechanical cushion for the CNS and a vehicle for distribution of nutrients and hormones through the CNS. CSF movement^12–16^ and CSF-ISF exchange^2,4,17–19^ during wakefulness, sleep and/or anesthesia recently have received particular interest for their implications on pathological states involving CSF. For instance, CSF together with ISF plays an essential role for the removal of solutes and metabolic wastes from the brain interstitium^1,3,4^. This waste removal plays a key role in a number of disease pathologies, such as Alzheimer’s^20^, which is the most common form of dementia contributing to ~ 60–70% of ~ 50 million dementia cases worldwide^21^, and is associated with the buildup of amyloid beta peptides^22,23^. Understanding the mechanisms driving CSF movement, and interventions that influence and enhance its resultant removal of waste products from the brain is therefore of high scientific and clinical impact.

CSF movement is driven by pressure changes in CNS vascular system due to cardiac pulsation (~ 1 Hz)^2,7,24–32^ and respiration (0.1–0.3 Hz)^12–16,33–38^, and is influenced by transient effects such as coughing^14,39–41^, and body posture^42,43^. A topic of current interest involving CSF dynamics is identifying the primary regulator(s) of CNS fluids or solute movement within subarachnoid spaces, ventricles, and deep brain parenchyma^2,7,12,14,36,37,44^. The major drivers of CSF flow implicated in recent studies are: (1) forced inspiration in humans^12^, (2) cardiac pulsation with some contribution from respiration in humans^37^, and (3) cardiac pulsation in rodents^7^. A few earlier studies particularly investigated respiratory-CSF dynamics during normal respiration using echo-planar imaging (EPI) (1.5T MRI)^34^, normal breathing and breath holding using dynamic EPI (1.5T MRI)^45^, normal and forced breathing using radial gradient-echo sequence (3T MRI)^12^. However, these studies measured CSF signal intensities in arbitrary units, and did not measure CSF directionality. A recent real-time multi slice EPI velocity phase contrast MRI (PCMRI) study^13^ showed directionality and magnitude of respiratory- and cardiac-driven CSF velocities in cm/s during a set of breathing patterns (normal, fast, and slow breathing), and breath holding (3T MRI). Recent works have also examined the magnitude, direction, and sensitivity of CSF movement to respiratory performances and locations^14–16,46,47^.

More recently, low-frequency oscillations of CSF (e.g., vasomotion; ~ < 0.1 Hz) including those during sleep have been of interest^9,48,49^. In a recent study conducted with subjects sleeping in a magnetic resonance imaging (MRI) scanner, Fultz and colleagues^9^ demonstrated that CSF flow oscillations during non-rapid eye movement (NREM) sleep were larger (5.52 dB) and slower (0.05 Hz vasomotion) compared to wakefulness (0.25 Hz respiratory), and suggested that changes in pulsatile CSF dynamics during sleep may alter the brain’s waste clearance due to increased mixing and diffusion^2,50^.

In short, CSF movement^15,16,51^ and removal of solutes^1,8,18,19,48,52–54^ from the brain is a topic of high clinical impact. Since pulsatile CSF dynamics is sensitive and synchronous to respiratory movements, we are interested in identifying potential integrative therapies such as yogic breathing to regulate CSF dynamics, which has not been reported before. To this end, we designed a study to investigate the impact of yogic breathing on pulsatile CSF dynamics.

### Yogic breathing

Mind–body approaches^55^ encompass a large and diverse group of therapies including yoga, meditation, Tai-Chi, Qi-Gong, and relaxation techniques. Among all, yoga has become one of the most popular integrative and complementary mind–body approaches^56^ of the 21th century for cultivating overall health and well-being. As a critical component of a traditional yoga practice, yogic breathing (*pranayama*^57^; the fourth limb of the — traditional — eight-limb path yoga practice^57,58^ consists of a variety of breathing techniques performed with mindful awareness, focused attention, and conscious control with a long-term goal of sustained mindful breathing pattern. One of the key principles of a regular yogic breathing practice is to make the breath slower, deeper, and rhythmical, which is associated with the self-regulatory mechanism and health-benefits^59–61^. Documented effects of slow breathing cover respiratory, cardiovascular, and cardiorespiratory autonomic nervous systems^60^. Further, through systematically paying attention to sensations of breathing, yogic breathing may be aimed to improve metacognitive awareness of mind–body connections and interoception, which is fundamentally a process linking brain and body^62^. For a systematic review of therapeutic benefits of yogic breathing see Jayawardena et al.^63^ One commonly studied mechanism for the health benefits of yogic breathing is its balancing effect on the autonomic nervous system through parasympathetic activation^60,64^. Since CSF is sensitive to respiratory dynamics^12–14,37^, we believe another potential mechanism for the benefits of yogic breathing is its influence on pulsatile CSF dynamics, which to date has not been reported.

We have recently developed a non-invasive real-time phase-contrast MRI (RT-PCMRI) approach^14^ that quantifies the influence of respiration and (harmonics of) cardiac pulsation on the (magnitude and direction of) instantaneous CSF velocities in absolute units [cm/s], which provides a unique opportunity to study the impact of yogic breathing on pulsatile CSF dynamics. We have utilized this RT-PCMRI in a recent randomized controlled trial (RCT) that aims to investigate effects of two separate 8-week yogic breathing interventions on pulsatile CSF dynamics. While the RCT aims to investigate the long-term impact of yogic breathing among novice practitioners, we herein present the pre-intervention baseline data, prior to randomization, to demonstrate the immediate impact of a set of slow and deep yogic breathing patterns on pulsatile CSF dynamics compared to spontaneous breathing by studying respiratory and cardiac-induced harmonic components of CSF. Briefly, we computed instantaneous-CSF (iCSF) velocities acquired with RT-PCMRI during spontaneous breathing and four yogic breathing practices (for a total of five breathing conditions). We then separated iCSF into three components: respiratory (rCSF), cardiac 1st (c_1_CSF) and 2nd harmonics (c_2_CSF), and rigorously tested the differences between spontaneous versus four yogic breathing conditions.

It is important to keep in mind that most studies of CSF dynamics have investigated the primary component of cardiac pulsation which is usually at the heart rate frequency. Cardiac-induced harmonics have been observed only in a few studies^65^ including in intracranial pressure (ICP) to assess pressure pulsatility, and recently in alligator CSF^66^ in the spinal canal and the cranial cavity. Further, several studies^67,68^ observed changes in CSF between individuals due to age and sex among other factors. Here, we provide cardiac-induced harmonics components of CSF movement as well as associations between demographic covariates (age, sex, and body mass index) and changes in CSF metrics during spontaneous versus yogic breathing conditions.

The primary goal of this pre-intervention baseline study is then two-fold: (1) to quantify and compare the immediate impact of four yogic breathing practices versus spontaneous breathing on (magnitude and direction of) velocities of iCSF, rCSF, c_1_CSF, and c_2_CSF, and (2) to quantify the relative contribution of rCSF versus c_1_CSF and c_2_CSF during each breathing condition to determine the primary regulator of CSF motion in all breathing conditions.

## Materials and methods

### Participants

**Figure 1:**
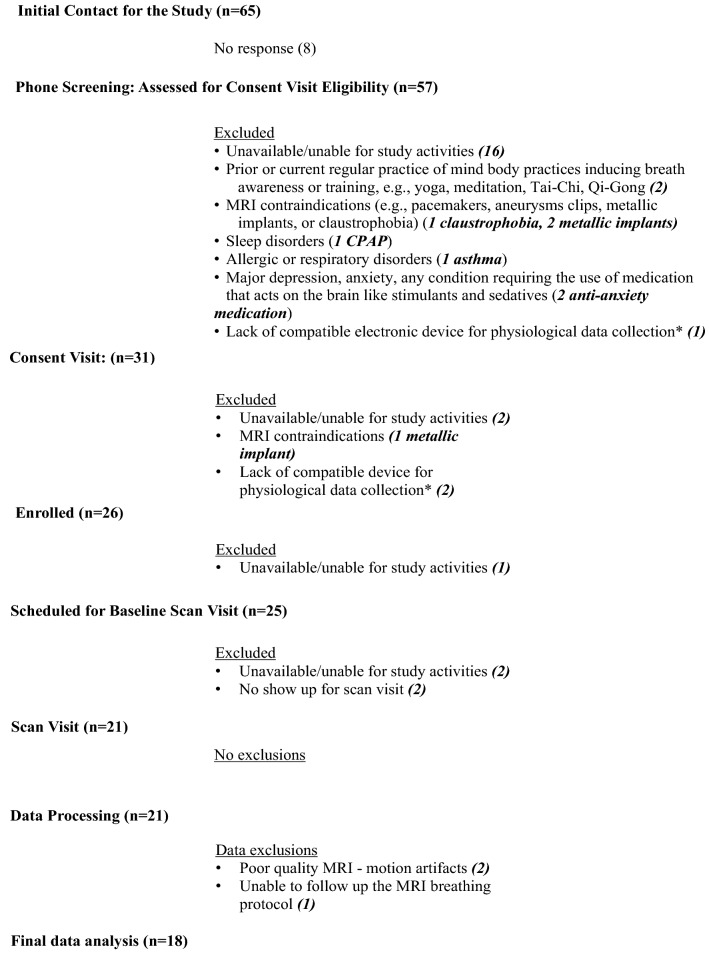
Study flow chart. *Our study utilized physiological data devices to objectively track participants’ home practice during the 8-week interventions. We excluded participants who did not have a compatible electronic device such as smartphone or tablet (see Table S1).

The study was approved by the Institutional Review Board of the Oregon Health & Science University (OHSU), and the full ongoing RCT was registered at the Clinicaltrials.gov (ID # NCT03858309). We received verbal and written informed consent from all study subjects prior to all study procedures. We recruited healthy participants from the Portland metropolitan area using OHSU’s study participation opportunities website, Oregon Center for Clinical and Translational Research Institute (OCTRI) research match for recruitment, flyers throughout the OHSU campus and communities in Portland, and social media (Facebook). We aimed to enroll participants 18 to 65 years of age who were able and available for study activities including undergoing non-invasive MRI scans, had no current or previous regular practice of mind–body therapies focusing on breath awareness and/or training (e.g., yoga, meditation, Tai-Chi, Qi-Gong), and were in good health without any history of neurological disorders, sleep disorders, respiratory disorders, problems with heart, circulatory system, and lungs. See Table S1 for a full list of RCT inclusion/exclusion criteria. Of the 65 participants contacted for the study, 57 were phone screened, 26 were enrolled, 21 completed the baseline procedures (September–October, 2019 at OHSU), and 18 were included in final baseline data analysis (N = 18, eight females, ten males; mean age: 34.9 ± 14 (SD) years; age range: 18–61 years). See Fig. 1 for the study flow chart, and Table 1 for the study group characteristics (N = 18).

**Table 1:** Study group characteristics.

*N*	Age range	Age in years Mean (SD)	Sex	BMI Mean (SD)	BP Mean (SD)	Race	Ethnicity
18	18–61	34.9 (14)	F, 8	24.2 (5.6)	Systolic, 123 (17)	1 African American	2 Hispanic or Latino
			M, 10		Diastolic, 75 (16)	4 Asian	16 Not Hispanic or Latino
						1 More than one race	
						1 Unknown	
						11 White	

### Experimental methodology

Each subject’s imaging visit lasted approximately 3-h including study instructions, 1-h MRI scans, and a set of questionnaires (as part of the RCT activities; not reported herein). Upon arrival for their imaging visit, we measured each subject’s temperature, blood pressure, and height and weight for body mass index (BMI). We then transitioned subjects to a mock scanner (0T room) for a ~ 30-min instruction for the breathing practices to be performed during the RT-PCMRI scans. The mock scanner room is designed to prepare research study subjects prior to entering the MRI Instrument suite, and is useful to help acclimate subjects to the enclosed space inside an MR Instrument. We first explained and demonstrated each breathing practice, then guided subjects to perform at their own pace first seated on a chair, and then in supine in the mock scanner to mimic the MRI environment.

#### MRI breathing protocol

We instructed subjects to perform the following breathing protocol first in the mock scanner for training purposes, and then in the MRI instrument during the ~ 1-min RT-PCMRI measurements, each to be collected twice with 30–60 s between consecutive measurements in the following order: (1) spontaneous breathing (SponB), (2) slow breathing (SlowB), (3) deep abdominal breathing (DAB), (4) deep diaphragmatic breathing (DDB) (5) deep chest breathing (DCB). See Table 2 for the MRI breathing protocol details.

**Table 2:** MRI breathing protocol.

MRI breathing protocol			
	Breathing pattern	Also known as	Performed
1	Spontaneous breathing (SponB)	Natural breathing, resting state breathing	With or without awareness on inhalation and exhalation without forcing to change the duration and/or the depth of the breath
2	Slow breathing (SlowB)	Slow rhythmic breathing	By consciously slowing down the breath with or without deepening the breath
			Choice of e.g., 3 to 5 counts* for each inhale/exhale
3	Deep abdominal breathing (DAB)	Belly breathing, lower breathing, part one of three-part breath	By consciously creating deep breaths in lower torso by expanding the lower abdomen with inhalation, and relaxing back to resting position with exhalation
			Choice of e.g., 3 to 5 counts* for each inhale/exhale
4	Deep diaphragmatic breathing (DDB)	Middle breathing, part two of three-part breath	By consciously creating deep breaths in middle torso by expanding lower ribs and diaphragm to the sides with inhalation and relaxing with exhalation
			Choice of e.g., 3 to 5 counts* for each inhale/exhale
5	Deep chest breathing (DCB)	Thoracic breathing, upper breathing, part three of three-part breath	By consciously creating deep breaths in upper torso performed by expanding the chest and the lungs to the sides, front and back with inhalation, and relaxing back to resting position with exhalation
			Choice of e.g., 3 to 5 counts* for each inhale/exhale

Of the five breathing techniques in the MRI breathing protocol, the last three deep breathing techniques (#3–5) together forms a specific yogic breathing called “*three-part breath;* Dirgha Pranayama”^70^. Typically, these three deep breathing practices are first performed in isolation for a few minutes for practitioners to gain ability to isolate the breathing and associated movement in lower, middle, and upper torso separately, with a long-term goal to improve the breathing apparatus and utilize the full capacity of the lungs. After several rounds of stand-alone performance in each part, practitioners then perform the *three-part breath* by combining all three parts during one breath: “During inhalation, beginning with expanding lower abdomen, smoothly moving the breath and expansion up to diaphragm, then to chest (all in one inhale); and during exhalation, beginning with contracting chest, smoothly moving the breath and contraction down to diaphragm, then to lower abdomen (all in one exhale)”, forming one repetition of a *three-part breath.*

#### Rationale for the MRI breathing protocol design

We chose breathing practices that were easily performed in supine in an MRI instrument without any constraints, and were less likely to cause head motion artifacts. We began with spontaneous breathing to observe each subject’s unique resting-state (natural) breathing patterns, and corresponding instantaneous CSF velocity waveforms. Since a key principle of a regular yogic breathing practice — that have been associated with health-benefits^59–61 ^— is to make the breath slower and deeper, we included slow and deep breathing practices that were likely to have immediate impact on pulsatile CSF motion, and create larger changes compared to spontaneous breathing in magnitude and frequency of pulsatile CSF motion based on our pilot studies and literature review^12–14^. Based on our investigations, we hypothesized slow breathing would create an impact in between spontaneous and deep breathing. Therefore, we utilized slow breathing in between spontaneous and deep breathing measurements. Three-part breath components were utilized in the order they are performed in a traditional yoga practice: abdominal, diaphragm and chest. We utilized this same order for each subject—instead of randomizing—to prevent any “carrying over” effect from slow and deep breathing patterns to spontaneous breathing. This would allow us (i) to compare each subject’s unique spontaneous versus yogic breathing patterns, and corresponding CSF velocity waveforms (ii) to then quantify changes in magnitude and frequency components of CSF for identifying the primary driving force of CSF (respiratory versus cardiac components) during spontaneous versus yogic breathing practices.

While we herein are interested in the impact of different deep breathing practices on CSF velocities — performed as in traditional yogic practices —, it is important to note the physiology of different breathing practices: a recent study^15^ provided the influence of deep abdominal versus deep thoracic breathing while noting the two-breathing patterns exerting different muscle groups. During abdominal breathing, the diaphragm is utilized as the inspiratory muscle, and changes in intrathoracic volume and intrathoracic pressure are greater during abdominal versus thoracic breathing. Particularly, more pronounced contracting of the diaphragm during abdominal breathing results in greater opening of costodiaphragmatic recess.

#### Breathing rate and depth

At the core of yogic breathing practices lies awareness and training of the breath. We designed our RCT yogic breathing interventions from Raja Yoga^69^ practices in the Himalayan Tradition, in which yogic breathing is suggested to be performed within each person’s own capacity for safety reasons, with inhale/exhale to be extended and expanded with caution through regular long-term practice. With that goal in mind, for the MRI breathing protocol, we specifically avoided enforcing any specific rate or depth for inhale/exhale other than giving a choice of rate (e.g., 3 to 5 counts with a count rate of 1/sec).

#### Subject preparation in the MR instrument

After being introduced to the breathing techniques in the mock scanner, we transitioned subjects to a 3T MRI instrument (MAGNETOM Prisma, Siemens Healthineers, Erlangen, Germany) for baseline data acquisitions using a 64-channel head and neck coil. We positioned subjects in supine, and provided them with (i) a bolster placed under the knees, (ii) foam pads under the elbows, (iii) pads around head and neck for comfort and minimizing motion artifacts, (iv) blankets for warmth, (v) a wireless finger pulse sensor (Siemens Health) for pulse data collection, and (vi) a respiration bellow (Siemens Health) for respiration data collection during the entire RT-PCMRI data acquisitions. We instructed subjects to lie still in supine during the entire data acquisition.

### Data acquisition

**Figure 2:**
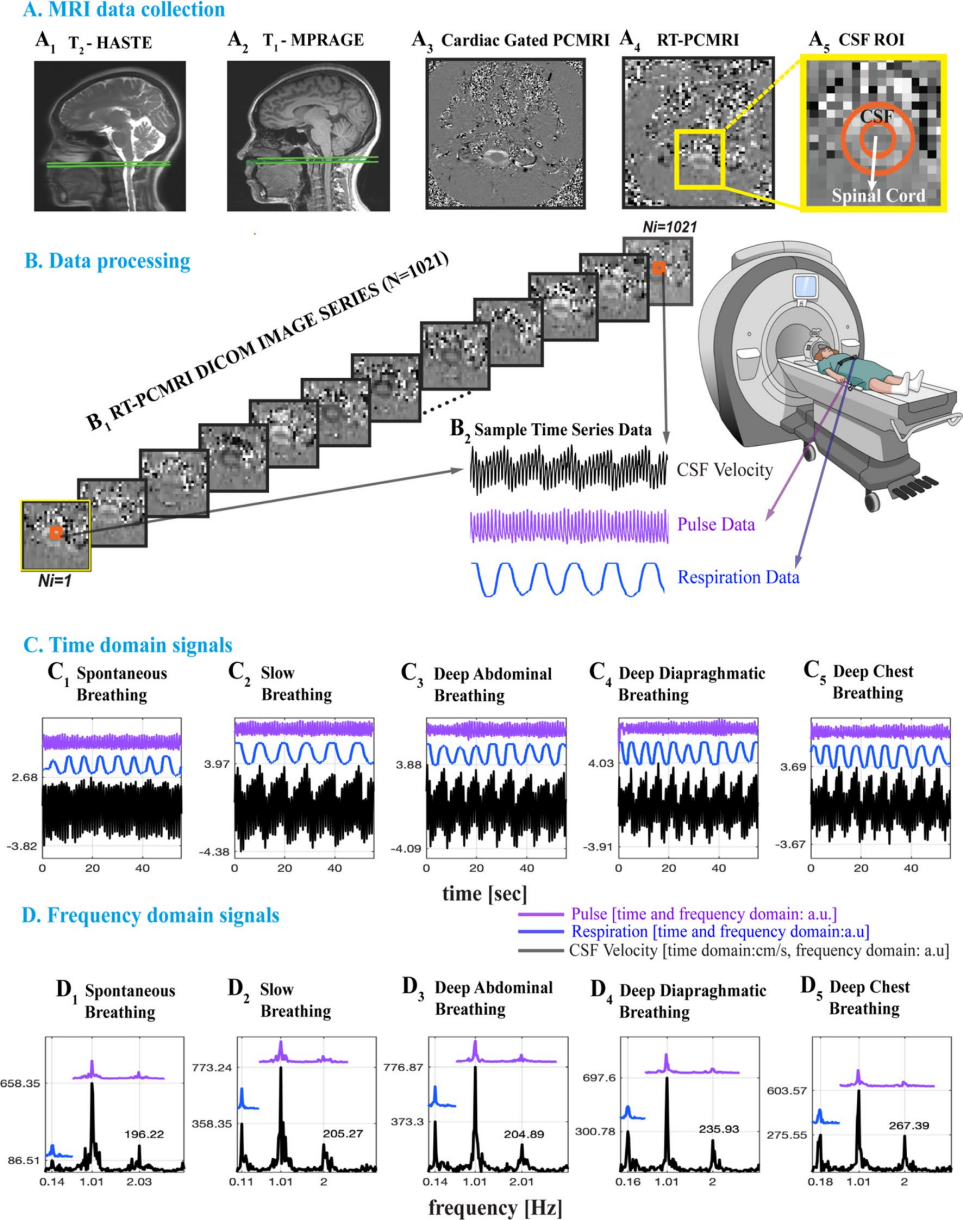
(**A**_**1–2**_) Sagittal anatomical MRI images showing the CSF measurement location at the level of foramen magnum (green lines) of a 37-year-old female. (**A**_**3**_) Axial images for cardiac gated PCMRI and RT-PCMRI velocity distribution. Cardiac-gated PCMRI is first collected for confirming CSF pulsation visibility prior to RT-PCMRI. ~ 1-min RT-PCMRI is then collected at the same location during five breathing patterns. (**A**_**5**_) A detailed image of region delineates the voxels of CSF and spinal cord (orange lines) and surrounding tissue. (**B**_1_) RT-PCMRI DICOM phase images (N = 1021) are collected for each breathing pattern repeated twice, resulting a total of 214,410 images processed for the n = 21 subjects, and 183,780 images utilized for the results of n = 18 subjects. (**B**_**2**_) Sample time series of CSF from single voxel RT-PCMRI (2.5 mm × 2.5 mm). Respiration and pulse data were simultaneously recorded, and temporally registered with the RT-PCMRI time series. (**C**–**D**) Time and frequency domain analysis of five breathing conditions: spontaneous breathing (SponB), slow breathing (SlowB), deep abdominal breathing (DAB), deep diaphragmatic breathing (DDB), and deep chest breathing (DCB) (with the last three forming a specific yogic breathing technique called *three-part breath*; see Table 2). When compared to SponB, both time domain maximum (positive; cranially directed) instantaneous CSF velocity values (in **C**), and peak respiration frequency amplitudes (in **D**) increase during SlowB, DAB, DDB, and DCB. Results are produced with MATLAB (https://www.mathworks.com/products/matlab.html) and presented using Adobe Illustrator (https://www.adobe.com/products/illustrator.html).

We utilized a 1-h data acquisition protocol, similar to our previous work^14^, consisting of anatomical MRI acquisitions, followed by simultaneous recordings of our previously established RT-PCMRI^14^ acquisition, respiration and finger pulse acquisitions. Briefly, for consistency across all subjects, we aimed to measure CSF at an angle perpendicular to the spinal cord at the level of the foramen magnum (FM) (Fig. 2A_1–2_ green lines). To determine the location of FM, we first collected anatomical MR images using a T_2_-weighted fast spin echo (HASTE, repetition time (TR) 1200 ms, echo time (TE) 80 ms; Fig. 2A_1_); a 3D T_1_-weighted gradient echo sequence (MPRAGE; TR 2300 ms; TE 2.32 ms; Fig. 2A_2_). We then acquired a cardiac gated PCMRI (TR 26.4 ms, TE 9.04 ms; Fig. 2A_3_) prior to RT-PCMRI to ensure proper slice location and angle for visibility of CSF pulsations, and that CSF was not obstructed. Upon confirming the pulsatile CSF motion (Fig. 2A_3_), we then acquired ~ 1-min RT-PCMRI (Fig. 2A_4–5_) at the same slice location and angle when subjects performed each of the breathing practices. RT-PCMRI sequence parameters included: velocity encoding value (VENC) 5 cm/s, temporal resolution ~ 55 ms, flip angle 30 degrees, matrix size 78 × 128, field of view (FOV) 196 × 323 mm (in-plane resolution ~ 2.5 × 2.5 mm), EPI factor 7, slice thickness 10 mm, TR 108.88 ms, TE 8.74 ms). RT-PCMRI has previously been described in detail^14^. During the RT-PCMRI acquisitions, we simultaneously collected respiration and pulse data with a sampling frequency of f_s_ = 400 Hz.

### Data processing

Each RT-PCMRI acquisition series produced 2042 images (1021 magnitude and 1021 phase, Fig. 2B_1_). In total (for N = 21 subjects, five breathing conditions (SponB, SlowB, DAB, DDB, DCB) each repeated twice) we have acquired 428,820 RT-PCMRI (magnitude and phase) images, and processed the needed 214,410 RT-PCMRI phase images for obtaining CSF velocity time series. We have developed a semi-automated protocol for post-processing all MRI DICOM images, and respiration and pulse data time series using MATLAB software packages [MATLAB, *R2019-2020*. Natick, Massachusetts: The MathWorks Inc.].

#### CSF ROI and velocity waveforms

**Figure 3:**
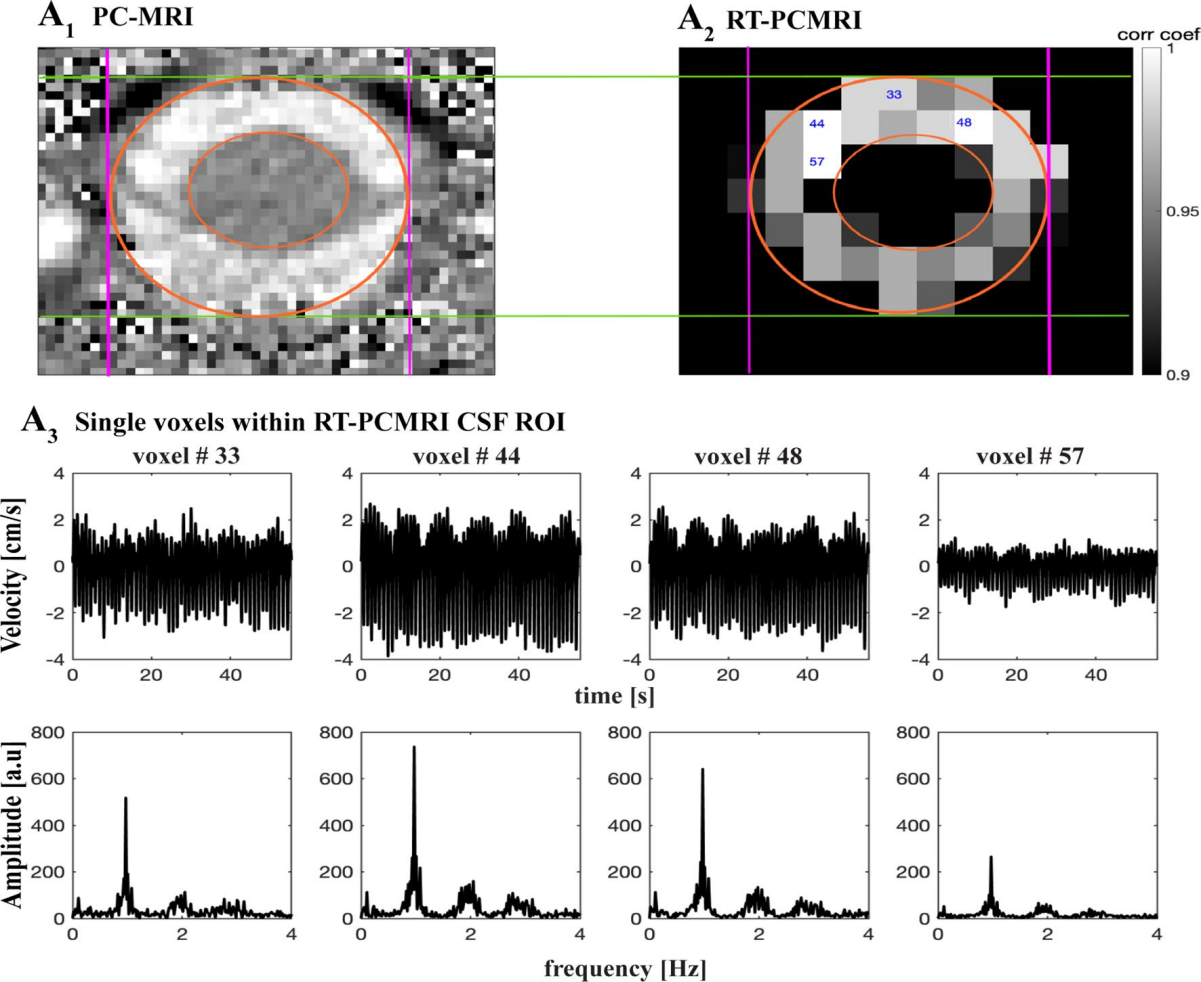
RT-PCMRI CSF ROI single voxel selection. (**A**) Sample conventional cardiac-gated PCMRI image (spatial resolution of 0.625 mm × 0.625 mm) showing the CSF region of interest (ROI) within orange circles. (**B**) Increasing temporal resolution for RT-PCMRI reduces the spatial resolution (2.5 mm × 2.5 mm). To capture true spatial velocity peak values, we implemented a 2-step process. Utilizing our previously developed correlation mapping technique, we first computed highly correlated CSF ROI voxels (greater than 0.7 correlation coefficient) — e.g., 33, 44, 48, 57 as labeled within each ROI. We then compared PCMRI image (**A**) with RT-PCMRI correlation map (**B**) to visually confirm the location of voxels. We are interested in maximum capacity of breathing impact on CSF. Anterior CSF velocities were usually greater than posterior velocities across our study population. Therefore, we have selected a single anterior voxel, within the CSF space and not contaminated by partial volume effects, and with greater velocity, which was usually among the highest correlated voxels obtained from the correlation mapping technique (greater than 0.9 correlation coefficient) such as voxel #44 for this sample. (**C**) For instance, while voxels #33, 48, and 57 are highly correlated one another, partial volume effects due to spinal cord or outside the subarachnoid space, the velocity waveforms and/or peak values are not truly preserved compared to voxel #44 which is within the CSF space. Results are produced with MATLAB (https://www.mathworks.com/products/matlab.html) and presented using Adobe Illustrator (https://www.adobe.com/products/illustrator.html).

A common method^30,32^ in conventional PCMRI studies to obtain CSF velocity time series is to average CSF across all voxels within the outlined region of interest (ROI), which may potentially cause spatial noise due to border zone partial volume effects^71^. Achieving a high temporal resolution for RT-PCMRI further may reduce the spatial resolution compared to conventional PCMRI, for which we previously developed a correlation mapping technique that allowed us to extract and average only highly correlated CSF voxels for an averaged CSF velocity time series. In this study, we are interested in obtaining and comparing true spatial and temporal CSF velocity values [cm/s] for each breathing practice. Therefore, to capture true spatial peak velocities to our best ability, we utilized a 2-step process to evaluate CSF velocity waveforms at a single voxel^9^ (Fig. 3). We first extracted highly correlated CSF voxels (greater than 0.7 correlation coefficient^14^) with our previously developed correlation mapping technique^14^, and then visually compared the CSF ROI voxels on RT-PCMRI images (spatial resolution of 2.5 × 2.5 mm) with the cardiac-gated PCMRI images (higher spatial resolution of 0.625 × 0.625 mm) to confirm the location of a single voxel of interest within CSF ROI. We are interested in maximum capacity of (participant) breathing impact on CSF. Anterior CSF velocities were usually larger than posterior velocities across our study population. We selected a single anterior voxel, within the CSF space, with greater velocity, which was usually among the highest correlated voxels (greater than 0.9 correlation coefficient) obtained from the correlation mapping technique. In addition, to confirm deep breathing practices did not cause artifacts in velocity values, potentially by B0 field changes in the head caused by the motion of torso during deep breathing, we computed CSF velocities in a set of voxels within static tissue, and confirmed there were no respiratory or cardiac frequency components (Fig. S1).

#### Time and frequency domain signals of interest

Previous studies^9,13,14,37,48^ reported vasomotion, respiration, and cardiac (1st harmonic) components of CSF signals. We observed (Fig. S2) higher order harmonics of cardiac pulsations in our preliminary analysis of frequency domain CSF velocity signals, which provides important information for determining the mechanisms, and their relative contribution to pulsatile CSF velocities. Having observed 1st and 2nd cardiac harmonics but not 3rd or 4th harmonics in all subjects, we have included 2nd cardiac harmonics in our analysis. In short, we are interested in four distinctive CSF velocity time series: instantaneous (iCSF), respiratory (rCSF), and cardiac 1st (c_1_CSF) and 2nd harmonics (c_2_CSF), which were below 4 Hz. To remove higher harmonics and high frequency signals, and investigate only the rCSF, respiration, c_1_CSF and c_2_CSF, we then low-pass filtered raw CSF velocity time series (see Fig. S3) using a 4th order Butterworth filter with a cut-off frequency of 4 Hz, which provided time domain iCSF velocity signals in Fig. 2C_1–5_. We then computed frequency domain signals using a fast Fourier transform (Fig. 2D_1–5_). See Fig. S4 for sufficient resolution of signals in Fig. 2C_1–5_, D_1–5_.

**Figure 4:**
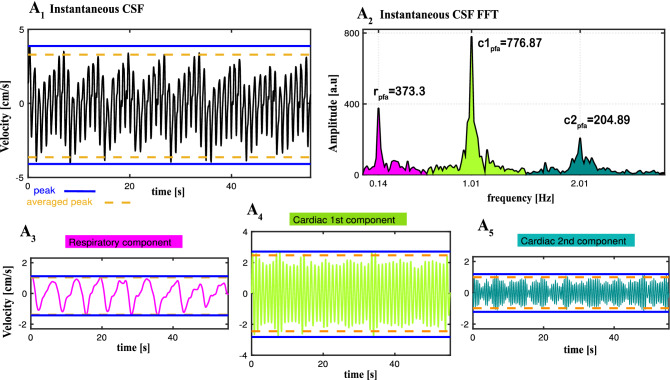
Separation of instantaneous CSF (iCSF) velocity waveforms (measured during deep abdominal breathing (DAB) for a 37 y–o female) into three components : respiratory (rCSF), cardiac 1st harmonic (c_1_CSF) and cardiac 2nd harmonic (c_2_CSF). (**A**_**1**_) Time domain iCSF velocity waveforms. (**A**_**2**_) Frequency domain iCSF obtained from Fast Fourier Transform (FFT) of iCSF velocity time series presenting peak frequency amplitudes for respiration (r_pfa_), cardiac 1st harmonic (c_1pfa_) and 2nd harmonic (c_2pfa_). We filtered iCSF within the bandwidth of each component — respiratory (magenta), cardiac 1st harmonic (light green) and 2nd harmonic (dark green) — in order to obtain and compare the characteristics of individual pulsatile velocity time series **(A**_**3**_) rCSF, (**A**_**4**_) c_1_CSF, and (**A**_**5**_) c_2_CSF. We computed maximum (positive; cranially directed) and minimum (negative; caudally directed) CSF velocity value in two ways: (i) peak value obtained as the highest and lowest point (blue solid line) during the entire time series, and (ii) averaged peak value (orange dash line) obtained by averaging the local maximums (and minimum, respectively) above a threshold set at 97.5th percentile (and 2.5th percentile, respectively). We used averaged peak values in statistical analysis to reduce temporal noise due to any transient events that may cause abrupt peaks (e.g., unexpected deep sigh) or lower peaks (indicating less than maximum capacity) caused by “participant fatigue” among novices during deep breathing conditions. Results are produced with MATLAB (https://www.mathworks.com/products/matlab.html) and presented using Adobe Illustrator (https://www.adobe.com/products/illustrator.html).

To separate and investigate rCSF, c_1_CSF and c_2_CSF (Fig. 4), we first computed frequency bands of each of the three components for each subject and breathing condition. This allowed us to take into consideration the individual and unique variations of frequency bands for accurate time domain velocity waveforms as well as frequency domain power calculations for each of the three components. We then filtered instantaneous CSF velocity waveforms (Fig. 4A_1_), using the individual frequency bands (Fig. 4A_2_), in (i) respiration frequency band of (estimated as typically f < ~ 0.6 Hz band), and (ii) cardiac 1st harmonic frequency band (estimated as typically ~ 0.6 < f < ~ 1.6 Hz band), and (iii) in cardiac 2nd harmonic frequency band (estimated as typically ~ 1.6 < f < ~ 2.7 Hz band) (Fig. 4A_3–5_). We repeated the above procedure for each breathing condition of each subject, and obtained time and frequency domain signals for all four distinctive CSF velocity waveforms.

In parallel, to confirm that estimated frequencies of CSF signals match with the physiological data, we filtered respiratory sensor and pulse sensor data in the same frequency band of respiratory and cardiac components of CSF velocity waveforms. For visualization purposes, we arbitrarily scaled the respiration and pulse data to compare with CSF velocity waveforms (Fig. 2C–D_1–5_). We confirmed the respiratory component in respiration data, and cardiac (1st and 2nd) harmonic components in pulse data (blue and purple lines in Fig. 2D_1–5_).

### CSF metrics

#### Time domain CSF metrics

We computed the following metrics from time domain CSF velocity waveforms iCSF, rCSF, c_1_CSF, c_2_CSF for each subject and breathing condition: (1) peak^13,14,37^ maximum (cranially directed value at the highest point) and peak minimum (caudally directed at the lowest point) during ~ 1-min time series; (2) averaged peak maximum and averaged peak minimum obtained from the average of local peak maximums (and peak minimums, resp.) above (and below, resp.) a threshold set at 97.5th percentile^72,73^ (and 2.5th percentile, resp.); (3) range^32^ of peak maximum to peak minimum; (4) range of averaged peak maximum to averaged peak minimum; (5) displacement computed from the integration of the CSF velocity time series [cm/s] and converted to [mm]. Lastly, we computed % change in these metrics, from SponB to SlowB, DAB, DDB, and DCB.

Note that the traditional method for computing cranially- and caudally-directed velocities are to compute peak maximum and peak minimum values. In addition to peak maximum and minimum, for this study, we also computed averaged peak maximum and averaged minimum values. See Fig. 4 blue and orange dash lines for a comparison of peak versus averaged peak values. Since our goal during each breathing condition is to capture true maximum capacity of CSF velocity, the use of averaged peak approach allowed us to reduce temporal noise caused by (i) random transient events that are not part of the regular breathing pattern (e.g., unexpected deep sigh) resulting in greater peak values, or (ii) “participant fatigue” experienced — among novice practitioners — while performing slow and/or deep breathing conditions resulting in lower peak values. It is possible for novice practitioners to experience “fatigue” resulting in brief pause and/or reduced capacity of respiratory movements due to novelty of the practices. The longer and more frequently novices perform these breathing practices, the less likely they would experience “fatigue” as in meditation and other mindfulness practices^74^.

#### Frequency domain CSF metrics

**Figure 5:**
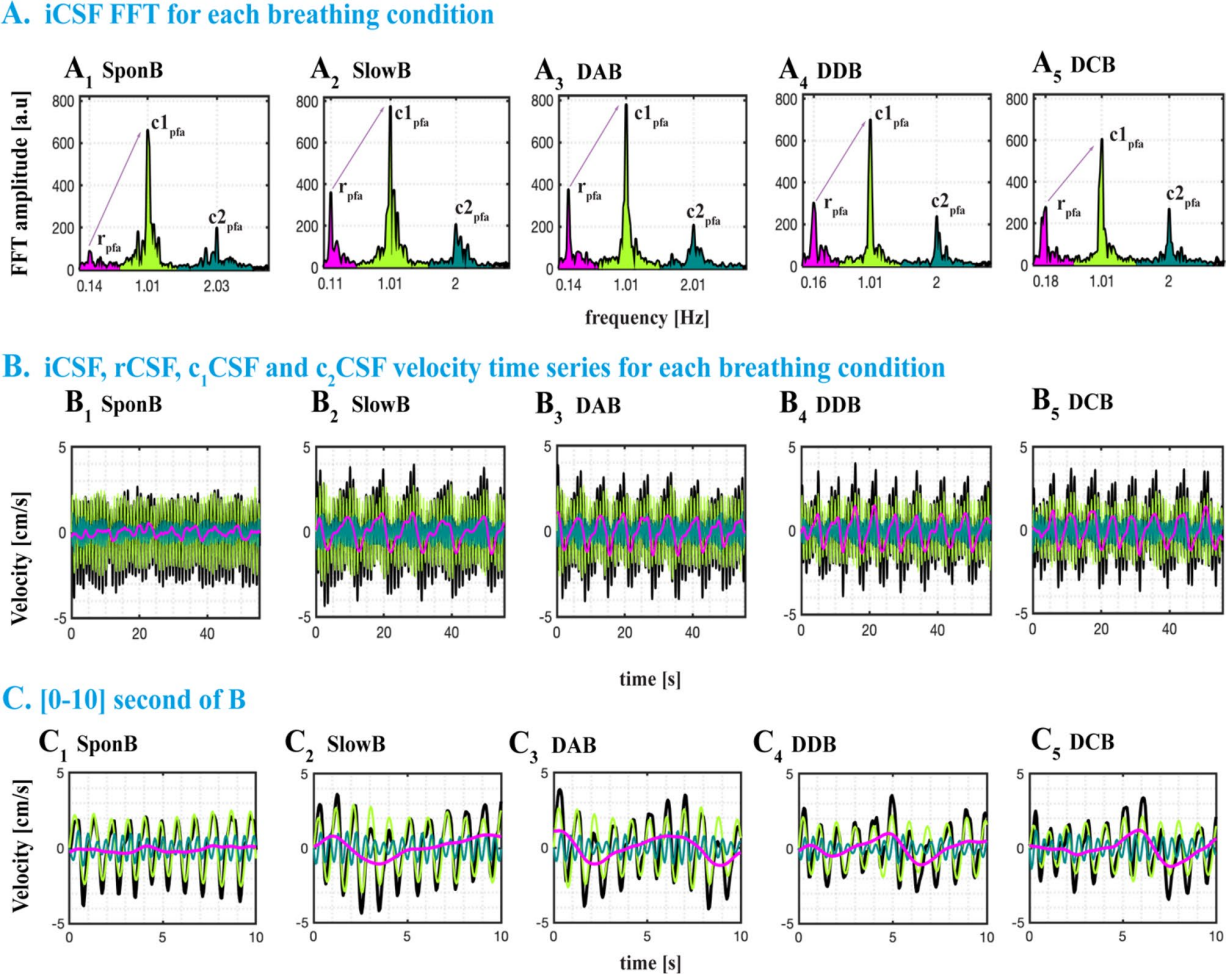
Sample datasets from a 37 y–o female presenting all four distinctive CSF signals (black: iCSF, magenta: rCSF, light green: c_1_CSF, dark green: c_2_CSF) during SponB, SlowB, DAB, DDB, and DCB. (**A**_**1–5**_) Frequency domain iCSF signals presenting the changes in peak frequencies [x-axis: r_pf_, c_1pf_, and c_2pf_; Hz] and peak frequency amplitudes [y-axis; r_pfa_, c_1pfa_, and c_2pfa_; a.u]. There was a decrease in respiration peak frequency during SlowB compared to SponB, and increase in respiration peak frequency amplitude r_pfa_ during all four yogic breathing techniques (**A**_**2–5**_) due to increased respiratory movement. We computed peak frequency amplitude ratios [r/c_1peak_] and [r/c_2peak_] (e.g., [r/c_1peak_] indicated by sample purple arrows) for observing the changes, and power ratios [r/c_1power_] and [r/c_2power_] for testing the relative contribution of each component to instantaneous CSF. (**B**_**1–5**_) Time domain CSF signals presenting an increase in both cranially directed iCSF and rCSF velocities during four yogic breathing techniques compared to SponB; with detailed waveforms presented in [0–10] s time window in (**C**_**1–5**_). Results are produced with MATLAB (https://www.mathworks.com/products/matlab.html) and presented using Adobe Illustrator (https://www.adobe.com/products/illustrator.html).

From the instantaneous CSF velocity waveforms, we have computed (1) peak frequencies, and (2) peak frequency amplitudes (Fig. 4A_2_). Additionally, to observe individual peak frequency amplitude ratio changes, we computed (3) a peak-to-peak frequency amplitude ratio [r/c_1peak_] and [r/c_2peak_] calculated as the ratio of rCSF peak frequency amplitude to the c_1_CSF, and c_2_CSF peak frequency amplitudes in the frequency domain (Fig. 5A_1–5_).

#### Relative contribution of rCSF, c_1_CSF, c_2_CSF signals

To compare the contribution of rCSF, c1CSF, and c2CSF and determine the primary regulatory force(s) for pulsatile CSF, we computed (1) estimated frequency band of rCSF, c1CSF, and c2CSF (Figs. 4A_2_, 5A_1–5_), (2) power of rCSF, c1CSF, and c2CSF, and (3) relative contribution^13,14,37^ of the respiration versus cardiac components by defining power ratio^14^ [r/c1power] and [r/c2power] calculated as the ratio of the power of the rCSF to the power of c1CSF and c2CSF, respectively.

To compute the power of rCSF, c1CSF, and c2CSF (defined^14^ as the integral of the square of the amplitude spectrum over the corresponding frequency band), we used trapezoidal numerical integration in frequency given by Eq. (1).

1 $${\int }_{a}^{b}S\left(f\right)df=\frac{b-a}{2N}\sum_{i=1}^{N}(S\left({f}_{i}\right)+S({f}_{i+1}))$$

where $$a$$ and $$b$$ are the first and last frequencies of the estimated frequency band (of signal of interest, e.g., rCSF, c_1_CSF, and c_2_CSF), ($$b-a)$$ is the frequency bandwidth, $$S$$ is the square of the signal amplitude spectrum, $$N$$ is the number of samples within the frequency band, and ($$b-a)/N$$ is the spacing between samples, i.e., $$df$$ frequency resolution. We then computed [r/c_1power_] as the ratio of rCSF power to the c_1_CSF power, and [r/c_2power_] as the ratio of rCSF power to the c_2_CSF power.

### Statistical analysis

To test the differences between spontaneous and four yogic breathing techniques, we used mean and standard deviation (SD) for the following time and frequency domain CSF metrics (i) averaged peak maximum and minimum values for iCSF, rCSF, c_1_CSF, c_2_CSF; (ii) range of iCSF averaged peak maximum and minimum values, (iii) iCSF displacement, (iv) peak frequencies, (v) peak frequency amplitudes, (vi) power values for rCSF, c_1_CSF, c_2_CSF, (vii) frequency peak-to-peak amplitude ratios, and (viii) power ratios for a total of 23 metrics (Table S2).

Independent statistical analysis was conducted by A.H. in R version 4.0.3 (R Core Team, Vienna, Austria). Though data were visually inspected, and extreme values were double checked based on percentiles (with a lower threshold of 5th percentile and a higher threshold of 95th percentile), all values remained in the data to preserve the—diversity of—differences in CSF velocities. Mixed effects linear regression models were built to analyze the associations between each outcome measure and each of the four experimental breathing conditions (SlowB, DAB, DDB, and DCB). Each model included a random subject effect to characterize within-person correlations over repeated measures. Normality assumptions of each model were checked by visual inspection of Q–Q Plots. For multiple comparisons, type I error rate was controlled by using the Benjamini–Yekutieli false detection rate (FDR) procedure^75^, with an overall FDR of 0.05, using the R function “p.adjust”. Unadjusted *p* values and FDR corrected *p* values are provided in Table S2, and *p* values mentioned within the text are FDR corrected *p* values.

In addition, associations between demographic covariates and outcomes were inspected visually, and tested for significance by Spearman’s rank-order correlation (continuous covariates) or t-test (dichotomous covariates) if a possible association was seen. We reported associations found to be significant in “Results” section. However, the regression models of the main analyses were not adjusted for these covariates in order to maintain consistency between models and so as not to overfit the models. Note this study was designed and powered to detect changes in CSF metrics after a yogic breathing intervention, rather than the pre-intervention baseline analysis that we present here. For full clinical trial sample size calculations, see Supplemental Materials.

### Ethics approval and consent to participate

The study was approved by the Institutional Review Board of the Oregon Health & Science University (OHSU). The clinical trial was registered at the ClinicalTrials.gov (ID # NCT03858309, February 28 2019, https://clinicaltrials.gov/ct2/show/NCT03858309). We received verbal and written informed consent from all study subjects prior to all study procedures. All the methods were carried out in accordance with relevant guidelines and regulations.

## Results

### Baseline group characteristics

Twenty-one subjects completed the baseline study activities. We processed 214,410 RT-PCMRI phase images, and removed datasets from final data analyses for three subjects; two subjects due to motion artifacts (e.g., compromised image quality), and one subject due to inability to follow the MRI breathing protocol. We then utilized 183,780 RT-PCMRI phase images from 18 participants for the final data analyses. See Fig. 1 for study flow chart, and Table 1 for baseline group characteristics.

**Figure 6:**
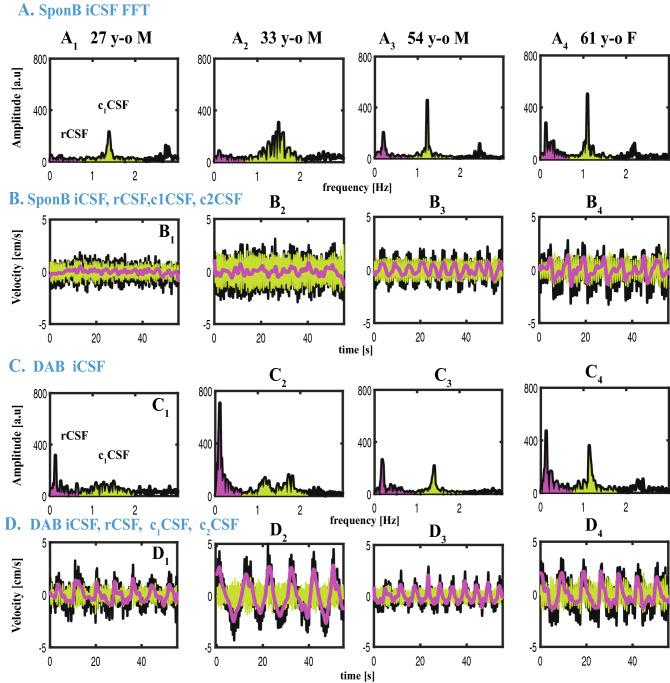
Datasets from four participants during SponB versus DAB to demonstrate the relative contribution of respiration versus cardiac 1st harmonic to pulsatile CSF, which was computed using a power analysis. (**A**) SponB (and **C** DAB) frequency domain iCSF signals. (**B**) (and **D**) Time domain velocity time series for iCSF (black), rCSF (magenta), c_1_CSF (lightgreen), and c_2_CSF (dark green). During SponB, across the 18 participants, cardiac pulsation was the major driver for pulsatile CSF, including four participants presented in (**A**–**B**). During DAB, while across the 18 participants, there was a comparable contribution of cardiac 1st harmonic and respiration, for these four participants, respiration was the major driver for pulsatile CSF due to significantly increased breathing depth resulting in increased [r/c_1power_] (**A** vs. **C**). Also see increase in cranially directed iCSF and rCSF peak velocities (**B** vs. **D**). Results are produced with MATLAB (https://www.mathworks.com/products/matlab.html) and presented using Adobe Illustrator (https://www.adobe.com/products/illustrator.html).

**Figure 7:**
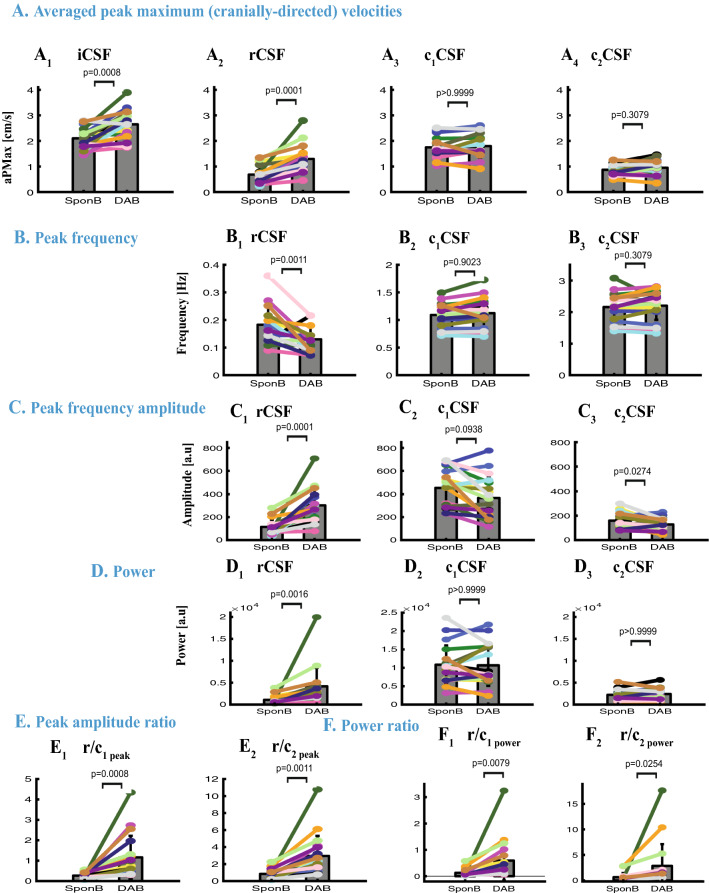
Comparison of time and frequency domain metrics used for SponB versus DAB. (**A**) Time domain: CSF velocity averaged peak (maximum cranially-directed) values for iCSF and rCSF significantly increased (*p* = 0.0008, and *p* = 0.0001), but not for c_1_CSF and c_2_CSF. (**B**–**F**) Frequency domain: (**B**) peak frequency for rCSF significantly decrease (*p* = 0.0011), with no significant changes for c_1_CSF and c_2_CSF. (**C**) Peak frequency amplitudes significantly increased for rCSF (*p* = 0.0001), and significantly decreased (*p* = 0.0274) for c_2_CSF with no significant changes for c_1_CSF. (**D**) Power for rCSF significantly increased (*p* = 0.0016) with no significant changes for c_1_CSF and c_2_CSF. (**E**) Peak amplitude ratios [r/c_1peak_] and [r/c_2peak_] significantly increased (*p* = 0.0008, and *p* = 0.0011). (**F**) Power ratios [r/c_1power_] and [r/c_2power_] significantly increased (0.0079, and *p* = 0.0254), with a greater contribution of respiration compared to cardiac 1st harmonic for four participants (as shown in Fig. 6). Results are produced with MATLAB (https://www.mathworks.com/products/matlab.html) and presented using Adobe Illustrator (https://www.adobe.com/products/illustrator.html).

We presented (i) sample datasets from a set of participants to demonstrate the changes in time domain and frequency domain CSF metrics during SponB versus yogic breathing (Figs. 2, 5, 6), (ii) statistical analysis results in Table S2 providing [adjusted mean difference, unadjusted *p* value, FDR *p* values], and (iii) group summary metrics (N = 18; all five breathing conditions) used for statistical analysis in Table S3–4, providing [mean, SD, %D]. Since we had 23 outcome measures to test the differences, we chose FDR which utilizes a Benjamini-Yekutieli algorithm, equipped to handle dependence between multiple outcome measures. We presented our main findings for SponB versus DAB in Fig. 7 (for all breathing conditions in Fig. S5), and will discuss group summary statistics in the following sections.

### Changes in time domain CSF metrics during SponB versus yogic breathing

For all subjects (N = 18; Table S2 and Table S3), during five breathing conditions, cranially-directed velocities (averaged peak maximum) were iCSF [2.10 to 2.65] cm/s, rCSF [0.68 to 1.29] cm/s, c_1_CSF [1.67 to 1.92] cm/s, and c_2_CSF [0.86 to 0.95] cm/s resulting in greater c_1_CSF velocities and comparable velocities for rCSF and c_2_CSF. (See Table S3 for mean and SD values during each breathing condition). When comparing the cranial iCSF velocities for SponB versus yogic breathing, we found an increase of 16–28% in cranial iCSF velocities during all yogic breathing conditions with statistical significance for SlowB (22%, *p* = 0.0287), DAB (28%, *p* = 0.0008; Fig. 7A_1_), DDB (23%, *p* = 0.0074), and an increase of 60–118% in cranial rCSF velocities during all yogic breathing conditions with statistical significance for DAB (118%, *p* = 0.0001, Fig. 7A_2_) and DDB (84%, *p* = 0.0074).

Caudally-directed velocities (averaged peak maximum) were iCSF [− 2.67 to − 3.03] cm/s, rCSF [− 0.67 to − 1.07] cm/s, c_1_CSF [− 1.69 to− 1.95] cm/s, and c_2_CSF [− 0.87 to − 0.95] cm/s resulting in greater c_1_CSF velocities and comparable velocities for rCSF and c_2_CSF. When comparing the caudal directed CSF velocities, we found an increase of 2–11% in caudal iCSF which did not reach statistical significance, and a decrease of 43–78% in caudal rCSF velocity with statistical significance for DAB (78%, *p* = 0.0014) and DDB (68%, *p* = 0.0074). There were no statistically significant findings for cranial (Fig. 7A_3–4_) and caudal c_1_CSF and c_2_CSF velocities, as well as iCSF displacement during SponB versus yogic breathing.

### Changes in frequency domain CSF metrics during SponB versus yogic breathing

When compared to SponB (Table S2 and Table S4), we found (1) a statistically significant decrease 18–42%; (*p* < 0.05) in estimated rCSF peak frequency (respiration rate) during all yogic breathing conditions with most significance for SlowB (42%, *p* < 0.0001) (see Fig. 7B_1_ for DAB), (2) an increase of 101–234% in rCSF peak frequency amplitude with statistical significance for SlowB (141%, *p* = 0.0287), DAB (234%, *p* = 0.0001, Fig. 7C_1_), and DDB (160%, *p* = 0.0172), (3) a decrease of 13–21% in c_2_CSF peak frequency amplitude with statistical significance for SlowB (20%, *p* = 0.0287), DAB (15%, *p* = 0.0274, Fig. 7C_3_), and DDB (21%, *p* = 0.0078). There were no statistically significant changes in peak frequency for c_1_CSF and c_2_CSF (Fig. 7B_2–3_), except an increase for DCB; 6%, *p* = 0.0496, and peak frequency amplitude for c_1_CSF (Fig. 7C_2_).

Additionally, we found an increase of 158–359% in peak amplitude ratio or rCSF to c_1_CSF [r/c_1peak_] with statistical significance for DAB (359%, *p* = 0.0008, Fig. 7E_1_), and an increase of 166–350% in [r/c_2peak_] with statistical significance for SlowB (223%, *p* = 0.0316), DAB (350%, *p* = 0.0011, Fig. 7E_2_), and DCB (265%, *p* = 0.0432).

### Relative contribution of rCSF, c_1_CSF, c_2_CSF during SponB versus yogic breathing

During yogic breathing compared to SponB (Table S2 and Table S4), we found an increase of 187–472% in rCSF power with statistical significance for DAB (472%, *p* = 0.0016, Fig. 7D_1_), and no statistically significant findings for c_1_CSF and c_2_CSF power (Fig. 7D_2–3_). We computed relative contribution of rCSF versus c_1_CSF and c_2_CSF using the power ratios [r/c_1power_] and [r/c_2power_]. Power ratio [r/c_1power_] for each breathing condition was [SponB; 0.13 ± 0.15], [SlowB; 0.29 ± 0.51], [DAB; 0.59 ± 0.78], [DDB; 0.43 ± 0.56] and [DCB; 0.40 ± 0.60] demonstrating cardiac 1st as major source of pulsatile CSF motion during SponB. There was an increase of 248–534% in [r/c_1power_] during yogic breathing compared to SponB, with statistical significance for DAB (534%, *p* = 0.0079, Fig. 7F_1_) when there was a comparable contribution of respiration and cardiac 1st harmonic to pulsatile CSF. For instance, four of the 18 participants (Fig. 6) presented greater respiratory power compared to cardiac 1st harmonic power during DAB versus SponB resulting in respiration as the major driver for pulsatile CSF during DAB for these four participants.

Power ratio [r/c_2power_] for each breathing condition was [SponB; 0.63 ± 0.81], [SlowB; 1.72 ± 3.52], [DAB; 2.85 ± 4.38], [DDB; 2.16 ± 2.96] and [DCB; 1.75 ± 2.39] demonstrating comparable contribution of respiration and cardiac 2nd harmonic during SponB. There was an increase of 234–589% in [r/c_2power_] during yogic breathing compared to SponB, with statistical significance for DAB (589%, *p* = 0.0254, Fig. 7F_2_).

### Covariates of age, sex, BMI

We tested associations between demographic covariates and outcomes during SponB versus yogic breathing. Under the DCB condition, there was a positive correlation between age and change scores (defined as change from the SponB condition) for cranial rCSF velocity (rho = 0.69, *p* < 0.001), rCSF frequency peak amplitude (rho = 0.75, *p* < 0.001), rCSF power (rho = 0.79, *p* < 0.001), and [r/c_1peak_; rho = 0.69, *p* = 0.001], [r/c_2peak_; rho = 0.67, *p* = 0.002], [r/c_1power_; rho = 0.80, *p* < 0.001]. [r/c_2power_; rho = 0.80, *p* < 0.001]. There was also an association between sex and c_2_CSF frequency peak amplitude for the change score between SponB to DAB (*p* = 0.009), and SponB to DCB (*p* = 0.048), with a mean increase in peak amplitude for females and a mean decrease for males.

In short, when compared to SponB, the main results were as follows; there was (1) a statistically significant decrease in respiration rate 18–42% during yogic breathing with most significance for SlowB (42%, *p* < 0.0001), (2) increase of 16–28% in cranially directed iCSF velocities with most statistical significance for DAB (28%, *p* = 0.0008), with no significance for DCB, (3) in parallel, an increase of 101–234% in rCSF peak frequency amplitude with most significance for DAB (234%, *p* = 0.0001), with no significance for DCB, (4) increase of 187–472% in rCSF power with statistical significance only for DAB (472%, *p* = 0.0016), (5) increase of 248–534% in [r/c_1power_] and 234–589% in r/c_2power_ with statistical significance only for DAB (534%, *p* = 0.0079, and 589%, *p* = 0.0254, resp.), (6) positive association between age and change scores from SponB to DCB.

## Discussion

We measured CSF velocities at the level of FM with a non-invasive RT-PCMRI approach, and found an immediate impact of four different types of yogic breathing techniques on pulsatile CSF velocities compared to spontaneous breathing. Results indicate the following findings (i) respiration rate significantly decreased during slow and deep yogic breathing techniques; (ii) cranial iCSF velocities and in parallel rCSF peak frequency amplitudes increased during yogic breathing with greatest effects in DAB, and with no significant effects for DCB, (iii) cardiac pulsation was the primary driving force for pulsatile CSF during all breathing conditions except DAB when there was a comparable contribution of respiration and cardiac 1st harmonic, and (iv) while there was a comparable contribution of respiration and cardiac 2nd harmonic during SponB, power ratio of respiration to cardiac 2nd harmonic increased during yogic breathing with greatest effects in DAB.

### Mechanics of respiratory CSF dynamics

Using a respiratory bellow, we collected respiration data simultaneously with the RT-PCMRI, thus confirming cranially-directed CSF during inhalation and caudally-directed CSF during exhalation. This result is in agreement with previous studies^24,39,76^ measuring CSF pressure recordings in response to respiratory changes, coughing and Valsalva maneuver, and non-invasive MRI studies^13,36,46^ investigating respiratory CSF velocities or flow volumes. Briefly, the transmission of venous pressure changes to the collapsible dura through thoracic and epidural veins lining the spine and around the vertebral column causes CSF movement in an ebb-and-flow manner. Lloyd et al.^16^ recently showed respiratory CSF flow is driven by lumbar and thoracic spinal pressures, and that reduced intrathoracic pressure during inspiration draws venous blood from the lumbar spine and cranium towards the thorax.

Of the four yogic breathing conditions we used in our study (SlowB, DAB, DDB, and DCB), the three of them (SlowB, DAB, and DDB) significantly increased iCSF velocities, with most pronounced effects observed during DAB with no significant change during DCB. The difference between abdominal and chest (thoracic) breathing we observed is aligned with previous reports indicating abdominal breathing is associated with larger respiratory pressure changes compared to thoracic breathing^15,77^. Aktas et al.^15^ recently demonstrated forced abdominal breathing—compared to forced thoracic breathing—has more pronounced effects on CSF movement within spinal subarachnoid space, resulting in upward net flow during both breathing patterns, whereas low flow rates were identified in the cerebral aqueduct in both breathing patterns. They concluded that abdominal breathing was associated with larger CSF flow due to a more pronounced contraction of the diaphragm compared to thoracic breathing. Furthermore, they suggested that changes in CSF dynamics were due to changes in intrathoracic and intraabdominal pressure being transmitted to the epidural space through the paravertebral venous plexus.

There were no statistically significant changes between SponB and DCB in our study, which may suggest our study population primarily consisted of natural chest breathers although other explanations are possible. While we observed significant increase in cranial directed iCSF velocities during SlowB, DAB, DDB, there were no changes in caudal iCSF velocities suggesting exhalation during spontaneous and yogic breathing in our study population was passive^16^.

### Primary sources of pulsatile CSF dynamics

The sources of pulsatile CSF velocity waveforms are cardiac pulsation, respiration and low frequency components such as vasomotion. Several studies recently examined primary regulator(s) of CSF movement and/or flow. For instance, (i) Dreha-Kulaczewski et al.^12^ presented CSF signal intensities (in arbitrary units) during forced inspiration, and suggested forced inspiration is the major driver of CSF while (ii) Takizawa et al.^37^ demonstrated velocities of cardiac-driven CSF at cerebral aqueduct were greater than respiratory-driven CSF, while displacement of respiratory-driven CSF was greater than cardiac-driven CSF, (iii) Mestre et al.^7^ more recently demonstrated cardiac pulsation is the primary regulator of CSF flow through perivascular spaces (PVSs) and is reduced in hypertension, and (iv) Fultz et al. demonstrated CSF flow is driven by vasomotion during sleep.

In our study, we presented respiratory and cardiac components of CSF while separating the cardiac 1st and 2nd harmonic components. During spontaneous breathing, we found that the cardiac 1st harmonic contributed greater power to pulsatile CSF velocities, with comparable contributions by respiration and the cardiac 2nd component. During yogic breathing, (i) the cardiac 1st harmonic contributed greater power to pulsatile CSF velocities, except during DAB, when there was a comparable contribution of respiration and cardiac 1st harmonic effect, and (ii) respiration contributed greater power compared to cardiac 2nd harmonic. During in all breathing conditions, we found a larger frequency amplitude of cardiac 1st versus 2nd harmonic, in agreement with earlier studies^65,78,79^ investigating ICP measures and a recent study^66^ investigating CSF dynamics of the American alligator. Studies^65^ observing cardiac-induced harmonics in ICP interpreted changes in brain pulsatility in the context of system compliance (of brain tissue, arterial, venous, and spinal thecal sac communication with brain through CSF spaces). Young et al.^66^ more recently (i) studied variations of pulsatile CSF in alligator in the spinal canal and the cranial cavity, (ii) found cardiac-induced harmonics in CSF (not above 3rd order), (iii) hypothesized the absence of higher harmonics could be related to the reptilian meninges and compliance. Taken together, higher harmonics of CSF provide important information for determining the mechanisms regulating CSF dynamics, and need to be investigated in further studies.

In addition, during yogic breathing compared to SponB, we observed an increase in respiration peak frequency amplitudes. Despite the significant increase in cranially directed iCSF velocities and in parallel in rCSF peak frequency amplitudes during SlowB, DAB, and DDB, cardiac pulsation was still the primary contributor (except during DAB), suggesting that the significant increase in CSF peak velocities or in rCSF peak frequency amplitudes did not necessarily mean that respiration was the major regulator for CSF dynamics. Thus, in future studies, we recommend doing a frequency domain power analysis to determine primary regulator(s) of pulsatile CSF dynamics. For instance, group summary results indicate that yogic breathwork increased both cranial directed CSF velocities and respiratory CSF peak amplitudes. However, only four individual subjects (Fig. 6) had greater respiratory power compared to cardiac power during DAB, suggesting (i) power contribution is critical, and (ii) respiration can be a major driver for pulsatile CSF dynamics depending on individual differences in breath “depth and location”. In short, even if CSF velocities may significantly increase with increased respiratory movement, if the increase in amplitude does not meet a certain threshold (e.g., not breathing deeply enough), it is the frequency of the driving mechanism, not the amplitude, that may have a more pronounced effect on driving CSF. As Williams^39^ pointed out cardiac pulsation transmits energy to the CSF, while wave propagation depends on pressure-induced differences in motion. Because venous blood and CSF are in equilibrium across venous membranes, venous changes create larger changes in CSF compared to arterial changes^80^. This could be the reason why Takizawa et al.^37^ observed greater cardiac — than respiratory-driven CSF velocities, and greater respiratory — than cardiac-driven CSF displacement.

Our study participants were naive to mind–body approaches, including breath awareness and breath training. During baseline data collection, most, if not all, of our study participants indicated that they were not familiar with any of the different deep breathing practices in our MRI protocol. Thus, the respiratory dynamics investigated in this baseline dataset provides only the immediate influence of yogic breathing in non-practitioners. In our ongoing interventional RCT study, we hypothesized that respiratory dynamics would be different in advanced practitioners, resulting in larger respiratory dynamics, and thus larger effects on CSF. In the RCT study, we will compare pre- and post-intervention respiratory dynamics utilizing the parameters as described herein to determine whether cardiac pulsation is still the primary driver of CSF post-intervention.

Differences in CSF dynamics between individuals, and across breathing conditions within individuals, suggest unique bio-individual characteristics of pulsatile CSF dynamics. Previous studies suggested changes in CSF between individuals could be due to age and sex^67,68^, vascularization^81^ and/or coupling between arterial inflow and venous outflow^82^. Based on our tests for associations between covariates (age, sex, and BMI), and changes in CSF metrics during SponB versus yogic breathing, we did not observe any significant change except a positive correlation between age and changes in SponB to DCB, in addition to a positive association with age and DCB condition alone. Due to small sample size in our study, we suspect these may be spurious findings. Future studies with larger sample size are needed to explore the associations for these covariates.

In short, we demonstrated that pulsatile CSF dynamics are highly sensitive and synchronous to respiratory characteristics such as rate, depth and location of respiratory movement, in agreement with previous studies^13,15,16,37^. We also provided the first comprehensive report studying a higher order (2nd) cardiac harmonic component in human CSF non-invasively during voluntarily controlled breathing conditions. Taken together, our results provide evidence for immediate modulation of pulsatile CSF dynamics with yogic breathing, and the importance of studying CSF dynamics in voluntarily controlled conditions to better understand mechanisms driving CSF.

### Implications

Recently, Fultz and colleagues^9^ conducted neuroimaging in human subjects during sleep by combining blood oxygen level–dependent functional magnetic resonance imaging (BOLD fMRI), electroencephalography (EEG), and CSF flow measurements, and demonstrated (i) CSF flow oscillations during non-rapid eye movement (NREM) sleep were larger and slower (0.05 Hz vasomotion) compared to wakefulness (0.25 Hz respiratory); and (ii) suggested changes in pulsatile CSF dynamics during sleep may alter brain’s waste clearance due to increased mixing and diffusion^2,50^. In our study, iCSF velocity waveforms were synchronous to breathing patterns, i.e., slower and larger during slow and deep yogic breathing practices compared to spontaneous breathing. Specifically, we found an increase of 16–28% in cranial iCSF velocities during yogic breathing. Because the entry of CSF along perivascular channels is critical for CSF-ISF exchange in rodents^18^, and because pulsatile CSF dynamics during sleep potentially may alter^9^ brain waste clearance due to increased mixing and diffusion, changes in pulsatile CSF dynamics due to yogic breathing could then be investigated for the removal of waste products in future studies.

In addition, changes in pulsatile CSF dynamics through yogic breathing could be beneficial for investigating intrathecal (IT) drug delivery and factors influencing IT drug transportation. For instance, using medical image-based computational fluid dynamics Hsu et al.^83^ studied drug transport as a function of frequency and magnitude of CSF pulsations during different heart rates and CSF stroke volumes. Both heart rate and CSF stroke volume influenced drug distribution in CSF presenting key factors for interpatient variability in drug distribution. We hypothesize that different breathing rates and CSF velocities via yogic breathing would impact peak concentration of drugs in CSF after injection through mixing and diffusion.

Further, our study may shed light on other components of yoga^84^, and other mind–body approaches^55^ with breathing awareness and/or training, such as mindfulness meditation^85^, mindfulness based stress reduction^86,87^, Tai-Chi^88^ or Qi-Gong^89^ in the context of CSF dynamics.

### Limitations and future studies

Our study is limited by the small sample size. The parent RCT was designed and powered to detect changes in CSF metrics after two breathing interventions, rather than the pre-intervention baseline analysis, and under the reasonable assumptions (please see power analysis for the full RCT in Supplementary Materials) ten subjects per arm would be sufficient to observe the proposed difference in the study arms. Future studies with a larger sample size would allow results to be more generalized across all five breathing patterns and varied demographics. The inherent challenges in MRI acquisition can lead to artifacts in MR images. Measurements with data artifacts were removed from the final analysis. Increased temporal resolution in our RT-PCMRI approach results in reduced spatial resolution, which we believe is handled through rigorous data processing methodology including semi-automated algorithm for extracting CSF signals, visually confirming CSF region of interest, and use of a single voxel approach. This approach eliminated partial volume effects, but limited CSF velocities within one voxel instead of entire cross section of CSF resulting in greater CSF velocities, and increased computational cost. Therefore, future work to develop high spatial and temporal resolution for continuous CSF measurements with analysis within the entire CSF region is needed. Despite these limitations, we have shown that our technique can detect and quantify CSF velocities around the spinal cord. To capture true temporal peak velocities and reduce noise due to transient events, we computed averaged peak velocities, which increased computational cost. We have collected pulse data with a finger pulse sensor, and respiration data with a respiration bellow. Future experimental methodology will include electrocardiography (ECG) measures for investigating the heart rate variability, and potential pressure sensors for measuring intrathoracic and abdominal pressures during yogic breathing techniques. Since our RCT focused on CSF dynamics, we have not investigated arterial and/or venous flow in this study.

Future investigations involving larger controlled studies of yogic breathing and/or other mind–body approaches will need to evaluate the effects of training of the breathing techniques on CSF measures. Investigations should: (i) use a larger sample size, (ii) study differences in age, sex, gender, race, activity levels, sleep quality, (iii) evaluate the influence of these covariates on pulsatile CSF magnitude and directionality along the spine and in the cranial cavity, (iv) study the coupling between CSF, arterial and venous flow, (v) utilize ECG, intrathoracic and abdominal pressure measurements in sync with MRI, and (vi) evaluate the effect of breathing induced changes in CSF on the brain’s waste clearance mechanism.

## Conclusions

To our knowledge, our study is the first report demonstrating the impact of a mind–body approach such as yogic breathing to modulate CSF dynamics, and comparing with spontaneous breathing. We investigated pulsatile CSF velocities during spontaneous versus yogic breathing practices (slow, deep abdominal, deep diaphragmatic and deep chest breathing) at the level of foramen magnum using a non-invasive MRI-based quantification in a set of healthy participants without current or previous regular practice of mind–body approaches. With rigorous testing, we demonstrated that the three yogic breathing patterns (slow, deep abdominal and deep diaphragmatic) immediately increased both cranially directed instantaneous CSF velocities and power of respiratory-driven CSF motion. We observed the most statistically significant effects during deep abdominal breathing. Cardiac pulsation was the primary driver of CSF motion during all breathing conditions except during deep abdominal breathing when there was a comparable contribution of respiration and cardiac 1st harmonic, which suggests respiration can be the primary driver for pulsatile CSF motion depending on individual differences in breathing technique. Further work is needed to investigate the influence of sustained yogic breathing training on pulsatile CSF dynamics for CNS health.

## Supplementary Information

Supplementary Information.

## Data Availability

The datasets generated for this study will be provided on a reasonable request to the corresponding author.
