# Supplementary material for: Immediate impact of yogic breathing on pulsatile cerebrospinal fluid dynamics
Source: Sci Rep. 2022 Jun 28;12:10894. doi: 10.1038/s41598-022-15034-8 (PMC9240010; doi:10.1038/s41598-022-15034-8)
Supplement: Supplementary file 1 — Supplementary Information. [file 41598_2022_15034_MOESM1_ESM.docx]

**Supplementary Materials for**

**Immediate Impact of Yogic Breathing on Pulsatile Cerebrospinal Fluid Dynamics**

**Authors.** Selda Yildiz ^1*^, John Grinstead ^2^, Andrea Hildebrand ^3^, John Oshinski ^4^, William D. Rooney ^1,5,6,7,8^, Miranda M. Lim ^1,6,9,10,11^, Barry Oken ^1,6^

**Affiliations.**

^1^Department of Neurology, Oregon Health & Science University, Portland, OR, 97239, USA

^2^Siemens Medical Solutions USA, Inc, Portland, OR, 97239, USA

^3^Biostatistics and Design Program, Oregon Health & Science University, Portland, OR, 97239, USA

^4^ Radiology & Imaging Sciences and Biomedical Engineering, Emory School of Medicine, Emory University, Atlanta, Georgia, 30322, USA

^5^Advanced Imaging Research Center, Oregon Health & Science University, Portland, OR, 97239, USA

^6^Department of Behavioral Neuroscience; Oregon Health & Science University, Portland, OR, 97239, USA

^7^Knight Cardiovascular Institute, Oregon Health & Science University, Portland, OR, 97239, USA

^8^Department of Biomedical Engineering, Oregon Health & Science University, Portland, OR, 97239, USA

^9^VA Portland Health Care System, Portland, OR, 97239, USA

^10^Department of Medicine, Division of Pulmonary and Critical Care Medicine; Oregon Health & Science University, Portland, OR, 97239, USA

^11^Oregon Institute of Occupational Health Sciences; Oregon Health & Science University, Portland, OR, 97239, USA

*Corresponding author. Selda Yildiz. Email: [yildiz@ohsu.edu](mailto:yildiz@ohsu.edu)

**This document includes:**

Supplementary text

Figures S1 to S5

Tables S1 to S5

**Materials and Methods**

| **Study Inclusion and Exclusion Criteria** | |
| --- | --- |
| **Inclusion Criteria** | - 18-65 years of age - Able to provide consent to be in the study - Willing and able to participate in the study activities - Able to lay supine - Able to walk 15 feet - Naive to mind-body practices, including breath awareness or training in yoga, meditation, Ta-Chi, Qi-Gong - Must have a compatible electronic device for collection of physiological data via a proprietary app* |
| **Exclusion Criteria** | - Inability to provide informed consent - MRI contraindications such as implanted medical devices or other non-removable metal (besides dental fillings), a larger body habitus (e.g., BMI > 30 or weight >250 pounds), claustrophobia - Need for muscle relaxants or anti-anxiety drugs in order to tolerate MRI - Any muscle or skeletal issues that would make it difficult to lie still in the supine position for the 1-hour MRI scan - History of neurological disorders such as stroke, brain hemorrhage, head trauma with loss of consciousness, multiple sclerosis, Parkinson’s disease, Huntington’s disease, Alzheimer’s disease - Sleep disorders - Allergic or respiratory disorders - Craniospinal disorders, e.g., Chiari Malformation. - Spinal injury - Major or uncontrolled psychiatric illness or major depression. - Any condition requiring the use of medication that acts on the brain such as stimulants, sedatives, antidepressants, antipsychotics, and anti-anxiety or anti-seizure medications - Active infection or uncontrolled significant systemic illnesses - Problems with the heart, circulatory system, or lungs such as asthma, chronic obstructive pulmonary disease, pulmonary edema, hypertension, cardiac arrhythmia, unstable angina pectoris, or symptomatic congestive heart failure - Smoking more than 5 cigarettes a day or drinking more than 3 alcoholic beverages a day - Current substance abuse, in drug rehabilitation, or regularly using marijuana - Women only: pregnant or nursing - On the day of MRI scan, fever >100.9^o^F |

**Table S1.** Study Inclusion and Exclusion Criteria. *Our study utilized physiological data devices to objectively track participants’ home practice during the 8-week interventions. We excluded participants who did not have a compatible electronic device such as smartphone or tablet.

**Confirming static tissue voxels are not impacted by deep breathing**


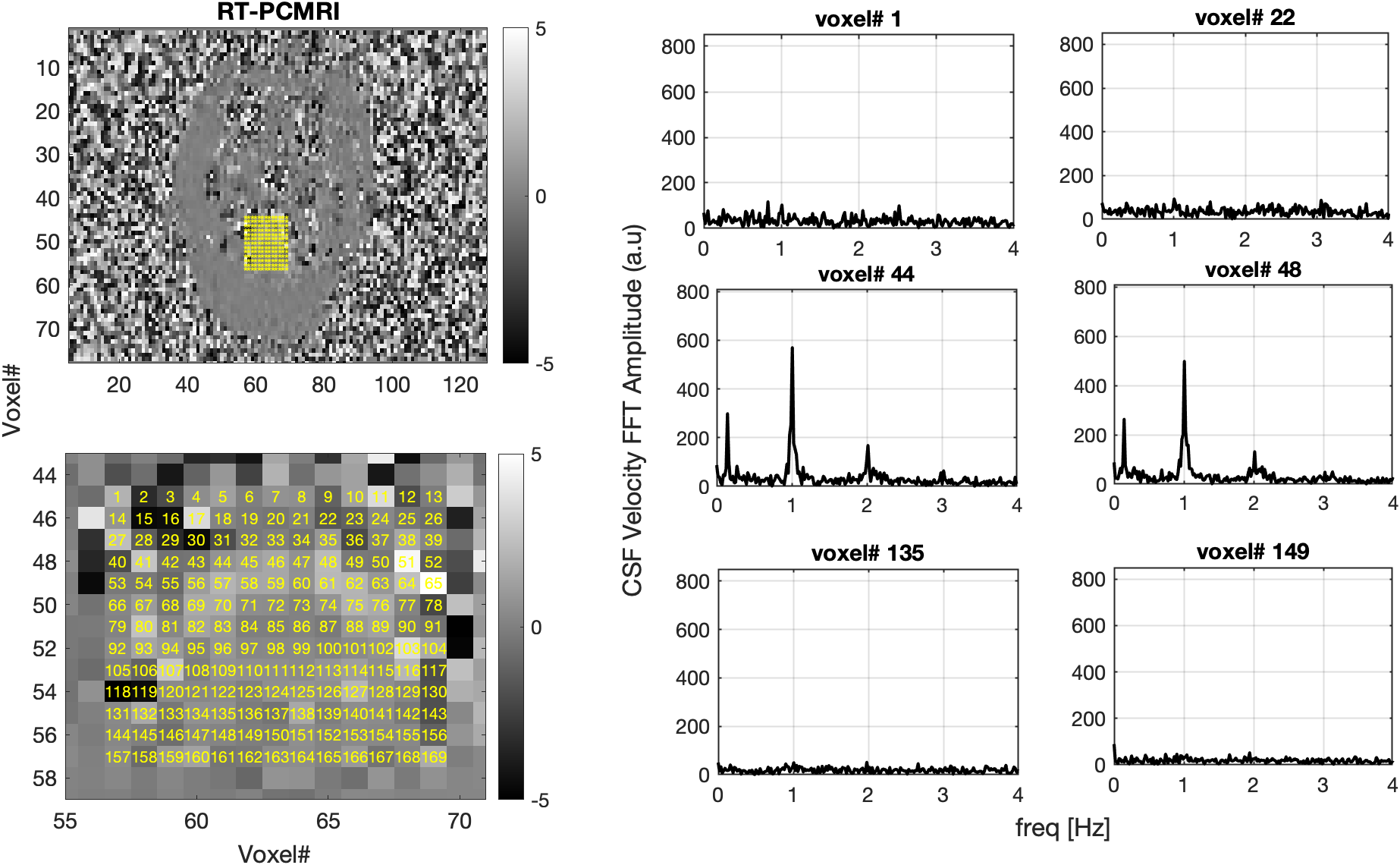


**Figure S1**. To confirm deep breathing practices did not cause any artifacts in CSF velocities, we computed frequency domain signals within static tissue versus CSF region of interest. While we observed peak amplitudes for respiratory and cardiac components in pixels with CSF, we did not observe any respiratory or cardiac components in static tissue.

**Observing cardiac harmonics in frequency domain CSF velocity signals**

**Figure S2.** Frequency components of CSF Velocities during spontaneous breathing (SponB) for the 18 participants. Previous studies reported vasomotion, respiration, and cardiac (1^st^ harmonic) components of CSF signals, but not cardiac 2^nd^ harmonic component. We observed higher order harmonics of cardiac pulsations (f > ~1.6 Hz) in our preliminary analysis of frequency domain CSF velocity signals. Having observed 1^st^ and 2^nd^ cardiac harmonics but not 3^rd^ or 4^th^ in all subjects, we have included 2^nd^ cardiac harmonics in our analysis because it provides critical information for determining the relative contribution of the underlying mechanisms regulating pulsatile CSF velocities.

**Raw versus filtered CSF velocity time series**


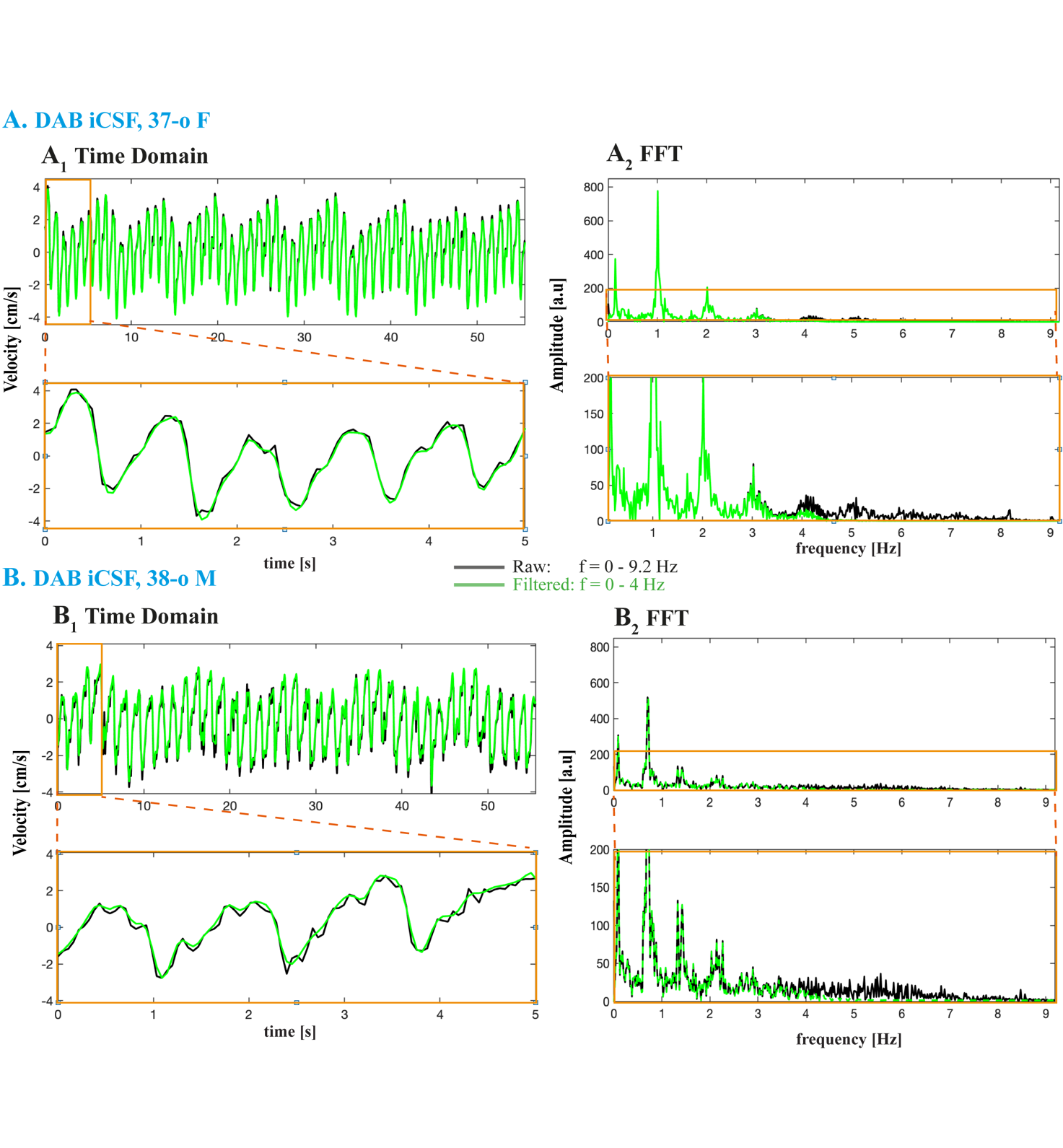


**Figure S3.** Sample deep abdominal breathing CSF velocity datasets: raw versus low-pass filtered data with a cut of frequency of 4 Hz for **A** 37-o female, **B** 38 y-o male. **A_1_** & **B_1_** time domain signals, and zoomed in for time [0-5] sec. **A_2_** & **B_2_** frequency domain signals, and zoomed in for magnitude of [0-200] a.u. In this study, we are interested respiration and first two harmonics of cardiac pulsation, all of which are below 4 Hz. To focus on signals of interest (respiration and first two cardiac harmonics), and to eliminate high frequency signals, we low-pass filtered raw CSF velocity time series using a 4^th^ order Butterworth filter with a cut of frequency of 4 Hz.

**Signals in Fig. 2C_1-5_ and Fig. 2D_1-5_ with sufficient resolution**


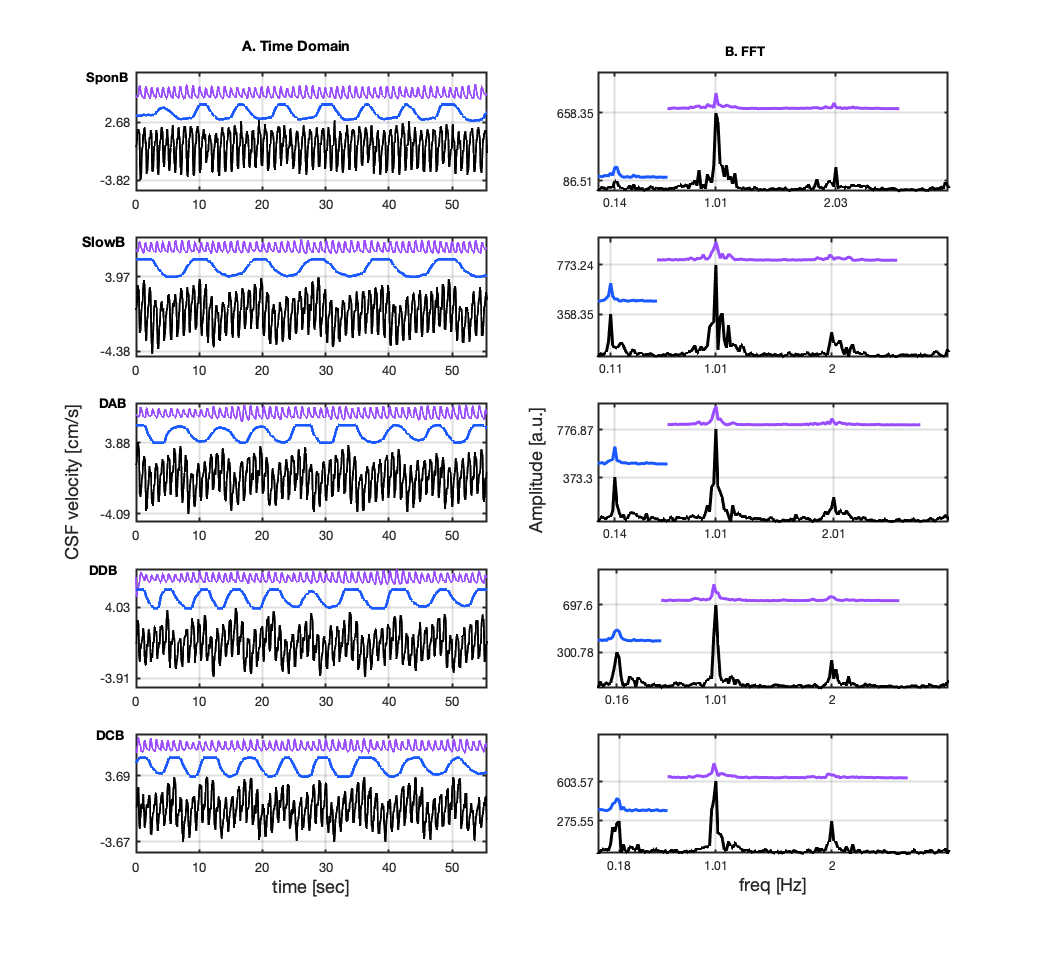


**Figure S4.** Time and frequency domain signals (**Fig. 2C_1-5_** and **Fig. 2D_1-5_**) presented for sufficient time resolution

**Power analysis for the full clinical trial**

This study is the pre-intervention baseline data analysis of a clinical trial, which was designed to understand the impact of two 8-week breathing interventions on CSF velocities. (1: active yogic breathing arm, and 2: slow breathing arm). The effects of the interventions on CSF variables are to be tested in a multivariate analysis of variance with repeated measures to compare pre- and post-intervention. Sample size estimates were derived from CSF velocity measures observed between yogic practitioners and non-practitioners (as obtained from our prior- unpublished- pilot study). In our previous study, practitioners presented with a 45-50% improvement in both cranial and caudal CSF flow (Cranial ΔCSF = 0.72 cm/s ± 0.62; Caudal ΔCSF = 0.58 cm/s ± 0.60). As an active intervention, it can be assumed that the increase in CSF would be marginally greater in a cohort previously untrained in yogic breathing practices. Furthermore, an active control group receiving slow breathing training would be anticipated to have an increase in CSF, albeit at an attenuated amount with an observed reduction in variance corresponding to the muted effect. We hypothesize that the yogic breathing would be an improvement over just slow breathing training, and YB intervention subjects, going through a regular practice of sophisticated breathing practices that have been previously shown to create large modulations in CSF flow, are expected to observe a greater increase in CSF shift somewhat larger than observed during pilot testing (ΔCSF=0.85, σ2=0.384). Based on these, it is reasonable to expect the active control would only see an improvement at 30% of the prior shift in CSF with half the variance found in the pilot (ΔCSF=0.225, σ2=0.192). Under these reasonable assumptions, 10 subjects per arm would be sufficient to observe the proposed difference in the study arms (cohort difference in ΔCSF = 0.625, d=1.16) at a one-tailed significance of α=0.05 with 80% power.

**Result**s

| **Statistical testing results for differences between SponB and four yogic breathing techniques.** | | | | | | | | | | | | |
| --- | --- | --- | --- | --- | --- | --- | --- | --- | --- | --- | --- | --- |
| **N=18** | **SlowB** | | | **DAB** | | | **DDB** | | | **DCB** | | |
|  | **adj.**  **mean diff.** | **unadj.**  **p** | **FDR**  **p** | **adj.**  **mean diff.** | **unadj.**  **p** | **FDR**  **p** | **adj.**  **mean diff.** | **unadj. p** | **FDR**  **p** | **adj.**  **mean diff.** | **unadj. p** | **FDR**  **p** |
| **iCSF**  **APMax** | 0.41 | 0.0013 | **0.0287** | 0.55 | <0.0001 | **0.0008** | 0.47 | 0.0003 | **0.0074** | 0.28 | 0.0241 | 0.3449 |
| **iCSF**  **APMin** | -0.29 | 0.0154 | 0.1650 | -0.24 | 0.0388 | 0.2380 | -0.13 | 0.2637 | >0.9999 | 0.07 | 0.5620 | >0.9999 |
| **iCSF**  **APMaxMin** | 0.70 | 0.0020 | **0.0316** | 0.79 | 0.0005 | **0.0051** | 0.59 | 0.0078 | 0.0744 | 0.22 | 0.3188 | >0.9999 |
| **rCSF**  **APMax** | 0.22 | 0.0635 | 0.3897 | 0.61 | <0.0001 | **0.0001** | 0.45 | 0.0003 | **0.0074** | 0.32 | 0.0095 | 0.2035 |
| **rCSF**  **APMin** | -0.24 | 0.0134 | 0.1648 | -0.39 | 0.0001 | **0.0014** | -0.38 | 0.0002 | **0.0074** | -0.19 | 0.0486 | 0.4176 |
| **c1CSF**  **APMax** | 0.17 | 0.0318 | 0.2480 | 0.05 | 0.5373 | >0.9999 | 0.04 | 0.5885 | >0.9999 | -0.08 | 0.3165 | >0.9999 |
| **c1CSF**  **APMin** | -0.18 | 0.0248 | 0.2129 | -0.05 | 0.5504 | >0.9999 | -0.02 | 0.8384 | >0.9999 | 0.08 | 0.3086 | >0.9999 |
| **C2CSF**  **APMax** | 0.06 | 0.1871 | 0.8484 | 0.08 | 0.0589 | 0.3079 | 0.08 | 0.0649 | 0.3857 | -0.01 | 0.8198 | >0.9999 |
| **C2CSF**  **APMin** | -0.06 | 0.1877 | 0.8484 | -0.08 | 0.0576 | 0.3079 | -0.08 | 0.0674 | 0.3857 | 0.01 | 0.8493 | >0.9999 |
| **iCSF**  **Disp** | -0.22 | 0.0628 | 0.3897 | -0.19 | 0.1115 | 0.5322 | -0.06 | 0.5797 | >0.9999 | 0.05 | 0.6671 | >0.9999 |
| **rCSF_f_** | -0.09 | <0.0001 | **<0.0001** | -0.05 | 0.0001 | **0.0011** | -0.05 | 0.0002 | **0.0074** | -0.04 | 0.0008 | **0.0496** |
| **c_1_CSF_f_** | -0.02 | 0.3495 | >0.9999 | 0.03 | 0.1996 | 0.9023 | 0.02 | 0.3698 | >0.9999 | 0.03 | 0.2431 | >0.9999 |
| **c_2_CSF_f_** | -0.04 | 0.2883 | >0.9999 | 0.07 | 0.0609 | 0.3079 | 0.11 | 0.0059 | 0.0629 | 0.13 | 0.0012 | **0.0496** |
| **rCSF_peak_** | 118.95 | 0.0012 | **0.0287** | 188.16 | <0.0001 | **0.0001** | 118.68 | 0.0012 | **0.0172** | 87.09 | 0.0156 | 0.2675 |
| **c_1_CSF_peak_** | -50.95 | 0.1502 | 0.7591 | -88.12 | 0.0142 | 0.0938 | -47.67 | 0.1778 | 0.9545 | -64.36 | 0.0704 | 0.5296 |
| **c_2_CSF_peak_** | -36.01 | 0.0010 | **0.0287** | -31.40 | 0.0038 | **0.0274** | -38.64 | 0.0005 | **0.0078** | -28.74 | 0.0078 | 0.2035 |
| **r/c_1peak_** | 0.48 | 0.0205 | 0.1952 | 0.89 | <0.0001 | **0.0008** | 0.45 | 0.0299 | 0.2134 | 0.42 | 0.0408 | 0.4118 |
| **r/c_2peak_** | 1.60 | 0.0022 | **0.0316** | 2.11 | 0.0001 | **0.0011** | 1.52 | 0.0035 | **0.0432** | 1.12 | 0.0294 | 0.3612 |
| **rCSF_power_** | 1424.75 | 0.0684 | 0.3917 | 3092.52 | 0.0001 | **0.0016** | 2066.65 | 0.0091 | 0.0780 | 1393.44 | 0.0746 | 0.5296 |
| **c_1_CSF_power_** | 1599.08 | 0.0469 | 0.3356 | -177.37 | 0.8230 | >0.9999 | -183.35 | 0.8172 | >0.9999 | -1403.29 | 0.0802 | 0.5296 |
| **c_2_CSF_power_** | 224.91 | 0.2550 | >0.9999 | 182.95 | 0.3537 | >0.9999 | 216.81 | 0.2724 | >0.9999 | -141.42 | 0.4729 | >0.9999 |
| **r/c_1power_** | 0.16 | 0.2341 | >0.9999 | 0.47 | 0.0009 | **0.0079** | 0.30 | 0.0273 | 0.2130 | 0.28 | 0.0431 | 0.4118 |
| **r/c_2power_** | 1.08 | 0.1407 | 0.7553 | 2.22 | 0.0032 | **0.0254** | 1.52 | 0.0396 | 0.2617 | 1.11 | 0.1293 | 0.7933 |

**Table S2.** Results of statistical testing, implemented for differences between SponB and four yogic breathing techniques. SponB: spontaneous breathing, SlowB: slow breathing, DAB: deep abdominal breathing, DDB: deep diaphragmatic breathing, DCB: deep chest breathing, iCSF: instantaneous-CSF, rCSF: respiratory-CSF, c_1_CSF: cardiac 1^st^ harmonic CSF, c_2_CSF: cardiac 2^nd^ harmonic CSF, APMax: averaged peak maximum, APMin: averaged peak minimum, APMaxMin: averaged peak maximum to averaged peak minimum, Disp: displacement, rCSFf: peak frequency of rCSF, c_1_CSFf: peak frequency of c_1_CSF, c_2_CSFf: peak frequency of c_2_CSF, rCSFpeak: peak frequency amplitude of rCSF, c_1_CSFpeak: peak frequency amplitude of c_1_CSF, c_2_CSFpeak: peak frequency amplitude of c_2_CSF, r/c_1peak_: peak frequency amplitude ratio of rCSF to c_1_CSF, r/c_2peak_: peak frequency amplitude ratio of rCSF to c_2_CSF, rCSF_power:_ power of rCSF, c_1_CSF_power_: power of c_1_CSF, c_2_CSF_power:_ power of c_2_CSF, r/c_1power_: power ratio of rCSF to c_1_CSF, r/c_2power_: power ratio of rCSF to c_2_CSF.

| **Time Domain CSF Metrics Assessed for All Breathing Conditions** | | | | | | |
| --- | --- | --- | --- | --- | --- | --- |
| **N=18** | | **SponB** | **SlowB** | **DAB** | **DDB** | **DCB** |
| **iCSF**  (0-4 Hz) | APMax [cm/s] | 2.10 (0.42) | 2.52 (0.53) | 2.65 (0.56) | 2.57 (0.60) | 2.39 (0.54) |
|  | *SponB %Δ* | | 21.55 (23.03) | 28.42 (28.74) | 23.33 (24.49) | 15.57 (26.22) |
|  | APMin [cm/s] | -2.74 (0.58) | -3.03 (0.64) | -2.98 (0.69) | -2.87 (0.68) | -2.67 (0.57) |
|  | *SponB %Δ* | | 11.28 (15.20) | 9.51 (16.72) | 5.60 (18.46) | -1.94 (10.43) |
|  | APMaxMin [cm/s] | 4.84 (0.92) | 5.54 (1.12) | 5.63 (1.11) | 5.44 (1.22) | 5.06 (1.03) |
|  | *SponB %Δ* | | 15.34 (16.93) | 17.38 (18.21) | 12.69 (17.36) | 5.23 (14.78) |
|  | Disp. [mm] | 0.041 (0.40) | -0.178 (0.35) | -0.146 (0.34) | -0.023 (0.41) | 0.091 (0.43) |
| **rCSF**  (0~0.6 Hz) | APMax [cm/s] | 0.68 (0.34) | 0.91 (0.45) | 1.29 (0.54) | 1.14 (0.58) | 1.00 (0.61) |
|  | *SponB %Δ* | | 60.27 (90.02) | 118.12 (113.24) | 83.68 (82.02) | 62.92 (90.67) |
|  | APMin [cm/s] | -0.67 (0.27) | -0.92 (0.5) | -1.07 (0.42) | -1.05 (0.46) | -0.87 (0.34) |
|  | *SponB %Δ* | | 52.90 (88.70) | 77.94 (81.30) | 68.14 (68.01) | 42.57 (62.35) |
|  | APMaxMin [cm/s] | 1.36 (0.57) | 1.82 (0.91) | 2.36 (0.88) | 2.19 (0.96) | 1.87 (0.89) |
|  | *SponB %Δ* | | 53 (85) | 96 (93) | 73 (72) | 50 (69) |
|  | Disp. (mm) | 0.014 (0.22) | -0.108 (0.29) | -0.056 (0.31) | -0.001 (0.24) | 0.073 (0.30) |
| **c1CSF**  (0.6~1.7 Hz) | APMax [cm/s] | 1.75 (0.40) | 1.92 (0.45) | 1.80 (0.46) | 1.79 (0.45) | 1.67 (0.39) |
|  | *SponB %Δ* | | 10.85 (16.33) | 3.18 (15.5) | 3.69 (18.78) | -3.72 (14.06) |
|  | APMin  [cm/s] | -1.77 (0.41) | -1.95 (0.48) | -1.81 (0.47) | -1.78 (0.46) | -1.69 (0.41) |
|  | *SponB %Δ* | | 10.85 (15.72) | 3.02 (15.79) | 1.72 (16.89) | -4.00 (13.14) |
|  | APMaxMin [cm/s] | 3.51 (0.81) | 3.86 (0.93) | 3.61 (0.93) | 3.57 (0.91) | 3.36 (0.80) |
|  | *SponB %Δ* | | 10.84 (15.94) | 3.08 (15.54) | 2.68 (17.76) | -3.88 (13.50) |
|  | Disp. [mm] | 0.019 (0.08) | 0.007 (0.06 | 0.004 (0.07) | 0.020 (0.09) | 0.025 (0.07) |
| **c2CSF**  (0.6~1.7 Hz) | APMax [cm/s] | 0.87 (0.21) | 0.93 (0.23) | 0.95 (0.28) | 0.95 (0.31) | 0.86 (0.22) |
|  | *SponB %Δ* | | 7.78 (19.31) | 9.71 (25.94) | 9.47 (27.10) | -0.09 (18.54) |
|  | APMin [cm/s] | -0.87 (0.21) | -0.93 (0.23) | -0.95 (0.29) | -0.95 (0.31) | -0.87 (0.23) |
|  | *SponB %Δ* | | 7.80 (19.37) | 9.63 (25.97) | 9.31 (27.21) | 0.02 (18.94) |
|  | APMaxMin [cm/s] | 1.75 (0.41) | 1.86 (0.46) | 1.91 (0.57) | 1.90 (0.61) | 1.73 (0.46) |
|  | *SponB %Δ* | | 7.80 (19.50) | 9.67 (25.93) | 9.39 (27.14) | -0.04 (18.72) |
|  | Disp. [mm] | -0.014 (0.03) | 0.004 (0.03) | -0.004 (0.02) | -0.006 (0.02) | -0.008 (0.02) |

**Table S3**. Time Domain CSF metrics (mean, SD, and %Δ compared to SponB) using averaged Peak Maximum/Minim values. SponB: Spontaneous breathing, SlowB: Slow breathing, DAB: deep abdominal breathing, DDB: deep diaphragmatic breathing, DCB: deep chest breathing, iCSF: instantaneous-CSF, rCSF: respiratory-CSF, c_1_CSF: 1^st^ cardiac harmonic component of CSF, c_2_CSF: 2^nd^ cardiac harmonic component of CSF, APMax: averaged peak maximum, APMin: averaged peak minimum, APMaxMin: averaged peak maximum to averaged peak minimum, Disp: displacement. All metrics -except APMaxMin for rCSF, c_2_CSF, and c_2_CSF- are used for statistical testing for the differences between SponB and four yogic breathing techniques.

Note that traditional method for computing cranially- and caudally-directed velocities are to compute peak maximum and peak minimum values. In addition to peak maximum and minimum (presented in **Table S5**), we computed averaged peak maximum and averaged minimum values. Since our goal during each breathing condition is to capture true maximum capacity of CSF velocity, the averaged peak values allowed us to reduce temporal noise caused by i) random transient events that are not part of the regular breathing pattern (e.g., unexpected deep sigh) resulting in greater peak values, or ii) “participant fatigue” experienced during performing slow and/or deep breathing conditions resulting in lower peak values.

| **Frequency Domain CSF Metrics Assessed for All Breathing Conditions** | | | | | | |
| --- | --- | --- | --- | --- | --- | --- |
| **N=18** | | **SponB** | **SlowB** | **DAB** | **DDB** | **DCB** |
| Peak  Freq.  [Hz] | rCSF_f_ | 0.18 (0.07) | 0.10 (0.02) | 0.13 (0.04) | 0.13 (0.05) | 0.14 (0.05) |
|  | *SponB %Δ* | | -42.44 (18.46) | -23.70 (31.22) | -21.76 (29.69) | -18.72 (33.10) |
|  | c_1_CSF_f_ | 1.09 (0.22) | 1.06 (0.25) | 1.12 (0.27) | 1.11 (0.28) | 1.12 (0.26) |
|  | *SponB %Δ* | | -2.11 (8.68) | 2.78 (8.58) | 1.78 (10.31) | 2.62 (8.70) |
|  | c_2_CSF_f_ | 2.13 (0.40) | 2.09 (0.38) | 2.20 (0.46) | 2.24 (0.49) | 2.26 (0.48) |
|  | *SponB %Δ* | | -1.60 (6.02) | 3.21 (8.30) | 4.85 (7.56) | 5.60 (8.77) |
| Peak  Amp.  [a.u.] | rCSF_peak_ | 114.65 ± 64.98 | 233.61 (177.10) | 302.81 (145.70) | 233.33 (122.37) | 201.74 (135.02) |
|  | *SponB %Δ* | | 140.62 (203.42) | 233.59 (244.68) | 160.39 (211.50) | 100.87 (140.32) |
|  | c_1_CSF_peak_ | 454.71 (162.77) | 403.77 (192.40) | 366.60 (182.68) | 407.04 (204.77) | 390.35 (208.72) |
|  | *SponB %Δ* | | -8.63 (33.31) | -19.70 (24.41) | -6.43 (21) | -10.42 (38.55) |
|  | c_2_CSF_peak_ | 159.42 (64.90) | 123.41 (50.34) | 128.02 (52.32) | 120.78 (52.58) | 130.68 (62.64) |
|  | *SponB %Δ* | | -20.39 (19.19) | -15.21 (32.40) | -20.62 (28.54) | -12.86 (36.75) |
| Peak  Amp.  Ratio | r/c_1peak_ | 0.27 (0.14) | 0.75 (0.83) | 1.16 (1.09) | 0.72 (0.64) | 0.69 (0.75) |
|  | *SponB %Δ* | | 193.21 (289.50) | 359.11 (394.92) | 191.82 (200.21) | 158.29 (176.33) |
|  | r/c_2peak_ | 0.84 (0.56) | 2.44 (3.06) | 2.95 (2.43) | 2.36 (2.07) | 1.96 (1.89) |
|  | *SponB %Δ* | | 223.12 (288.82) | 349.86 (412.26) | 265.23 (332.73) | 165.76 (185.06) |
| Freq.  Band  [Hz] | rCSF_fband_ | f < 0.60 (0.13) | f < 0.55 (0.10) | f < 0.60 (0.13) | f < 0.60 (0.15) | f < 0.63 (0.16) |
|  | c_1_CSF_fband_ | 0.60 (0.13)  < f <  1.65 (0.33) | 0.55 (0.10)  < f <  1.63 (0.30) | 0.60 (0.13)  < f <  1.66 (0.33) | 0.60 (0.15)  < f <  1.69 (0.35) | 0.63 (0.16)  < f <  1.70 (0.34) |
|  | c_2_CSF_fband_ | 1.65 (0.33)  < f <  2.70 (0.56) | 1.63 (0.30)  < f <  2.65 (0.56) | 1.66 (0.33)  < f <  2.82 (0.59) | 1.69 (0.35)  < f <  2.78 (0.60) | 1.70 (0.34)  < f <  2.70 (0.58) |
| Power  [a.u.] | rCSF_power_ | 1075.26 (975.60) | 2500.00  (3457.83) | 4167.78  (4351.75) | 3141.90  (3054.91) | 2468.69 (2811.90) |
|  | *SponB %Δ* | | 229.62  (395.49) | 471.51  (562.65) | 269.51  (314.29) | 187.36  (241.35) |
|  | c_1_CSF_power_ | 10843.7 (5369.9) | 12442.7  (6417.3) | 10689.4 (5713.3) | 10660.3  (6599.1) | 9440.3  (4700.4) |
|  | *SponB %Δ* | | -0.63 (7.59) | 1.34 (9.60) | 2.96 (13.29) | 3.72 (10.83) |
|  | c_2_CSF_power_ | 2224.13 (1159.82) | 2449.03  (1279.45) | 2407.07  (1379.31) | 2440.93  (1675.36) | 2082.70 (1176.44) |
|  | *SponB %Δ* | | 14.88 (35.51) | 11.27 (47.05) | 9.62 (48.13) | -4.19 (34.42) |
| Power Ratio | r/c_1power_ | 0.13 ± 0.15 | 0.29 (0.51) | 0.59 (0.78) | 0.43 (0.56) | 0.40 (0.60) |
|  | *SponB %Δ* | | 247.46 (617.84) | 533.68 (866.40) | 294.27 (348.82) | 249.35 (299.46) |
|  | r/c_2power_ | 0.63 ± 0.81 | 1.72 (3.52) | 2.85 (4.38) | 2.16 (2.96) | 1.75 (2.39) |
|  | *SponB %Δ* | | 324.26 (974.70) | 589.40 (1153) | 327.78 (513.69) | 234.62 (319.57) |

**Table S4.** Frequency Domain CSF metrics (mean, SD, and %Δ compared to SponB). SponB: spontaneous breathing, SlowB: slow breathing, DAB: seep abdominal breathing, DDB: deep diaphragmatic breathing, DCB: deep chest breathing, rCSF: respiratory-CSF, c_1_CSF: 1^st^ cardiac harmonic component of CSF, c_2_CSF: 2^nd^ cardiac harmonic component of CSF, APMax: averaged peak maximum, APMin: averaged peak minimum, APMaxMin: averaged peak maximum to averaged peak minimum, Disp: displacement, rCSF_f_: peak frequency of rCSF, c_1_CSF_f_: peak frequency of c_1_CSF, c_2_CSF_f_: peak frequency of c_2_CSF, rCSF_peak_: peak frequency amplitude of rCSF, c_1_CSF_peak_: peak frequency amplitude of c_1_CSF, c_2_CSF_peak_: peak frequency amplitude of c_2_CSF, r/c_1peak_: peak frequency amplitude ratio of rCSF to c_1_CSF, r/c_2peak_: peak frequency amplitude ratio of rCSF to c_2_CSF, rCSF_power_: power of rCSF, c_1_CSF_power_: power of c_1_CSF, c_2_CSF_power_: power of c_2_CSF, r/c_1power_: power ratio of rCSF to c_1_CSF, r/c_2power_: power ratio of rCSF to c_2_CSF. All metrics -except frequency band- are used for statistical testing for the differences between SponB and four yogic breathing techniques.


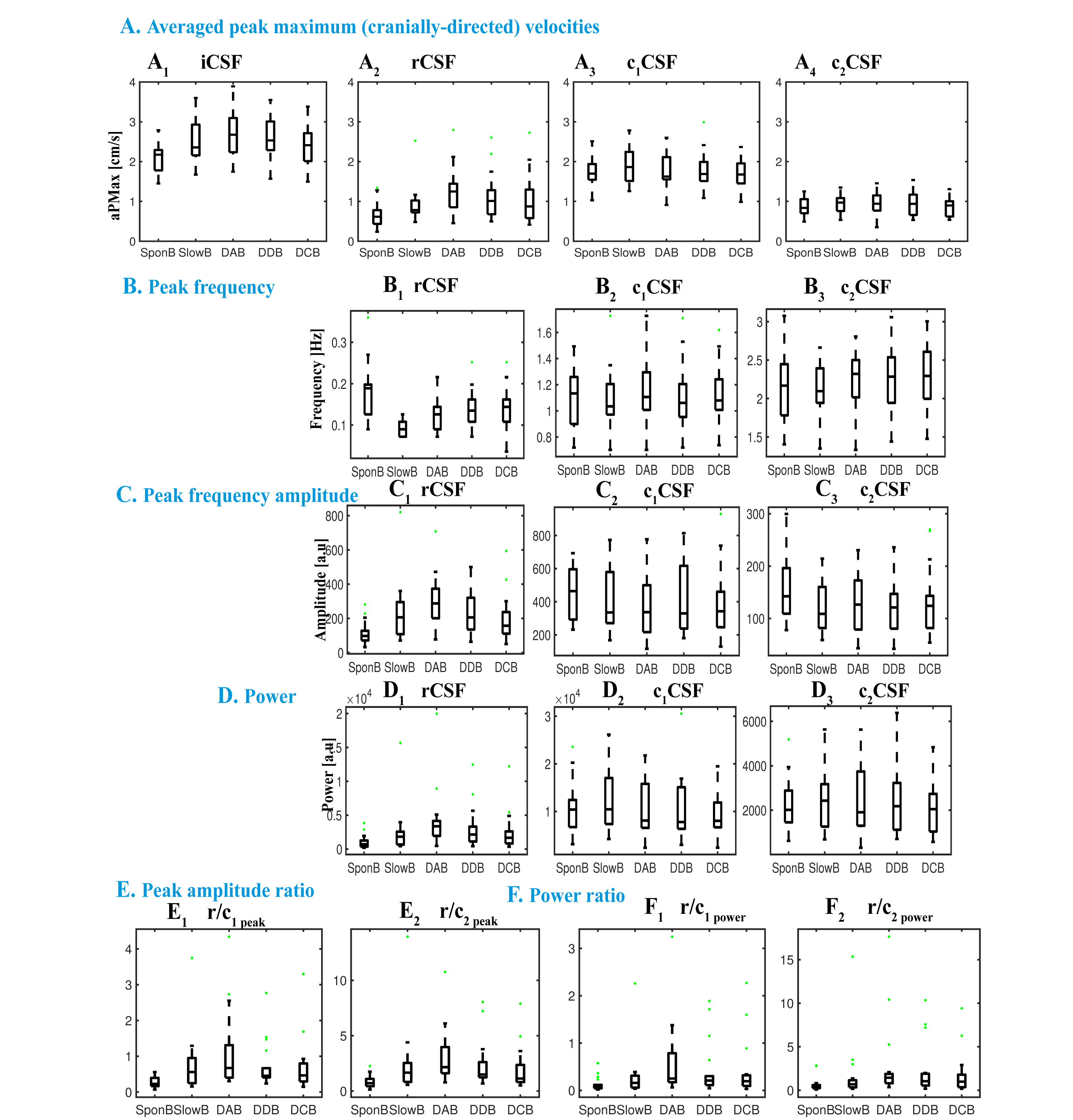
**Visualization of time and frequency domain metrics for all breathing conditions**

**Figure S5.** Time and Frequency Domain CSF metrics for all breathing conditions: SponB, SlowB, DAB, DDB, and DCB.

| **Time Domain: Peak Maximum, Peak Minimum, and Peak Maximum to Peak Minimum Assessed for All Breathing Conditions** | | | | | | |
| --- | --- | --- | --- | --- | --- | --- |
| **N=18** | | **SponB** | **SlowB** | **DAB** | **DDB** | **DCB** |
| **iCSF**  (0-4 Hz) | PMax  [cm/s] | 2.61 (0.53) | 3.06 (0.69) | 3.23 (0.70) | 3.11 (0.65) | 2.90 (0.61) |
|  | *SponB %Δ* | | 19.09 (23.11) | 27.12 (30.41) | 21.35 (23.97) | 14.26 (28.37) |
|  | PMin  [cm/s] | -3.23 (0.62) | -3.63 (0.78) | -3.55 (0.81) | -3.50 (0.82) | -3.23 (0.63) |
|  | *SponB %Δ* | | 13.12 (19.12) | 10.56 (20.33) | 9.68 (24.94) | 0.70 (12.06) |
|  | PMaxMin [cm/s] | 5.84 (1.08) | 6.69 (1.41) | 6.78 (1.30) | 6.61 (1.37) | 6.12 (1.09) |
|  | *%Δ (SponB)* | | 15.30 (18.40) | 17.76 (21.69) | 14.43 (21.47) | 6.33 (17.05) |
| **rCSF**  (0~0.6 Hz) | PMax  [cm/s] | 0.87 (0.45) | 1.12 (0.50) | 1.49 (0.57) | 1.26 (0.65) | 1.10 (0.66) |
|  | *SponB %Δ* | | 59.88 (91.29) | 99.58 (97.06) | 64.40 (76.88) | 47.73 (91.27) |
|  | PMin  [cm/s] | -0.85 (0.35) | -1.13 (0.65) | -1.30 (0.44) | -1.22 (0.42) | -1.11 (0.47) |
|  | *SponB %Δ* | | 65.79 (157.23) | 83.03 (116.76) | 64.54 (76.13) | 58.73 (107.36) |
|  | PMaxMin [cm/s] | 1.72 (0.70) | 2.25 (0.10) | 2.78 (0.82) | 2.48 (0.99) | 2.21 (0.98) |
|  | %Δ (SponB) | | 54.48 (108.75) | 87.26 (100.41) | 58.94 (70.53) | 46.55 (85.88) |
| **c1CSF**  (0.6 ~1.7 Hz) | PMax  [cm/s] | 2.02 (0.46) | 2.21 (0.52) | 2.09 (0.51) | 2.13 (0.51) | 1.97 (0.43) |
|  | *SponB %Δ* | | 11.25 (19.33) | 4.65 (19.84) | 7.97 (28.09) | -0. 89 (17.31) |
|  | PMin  [cm/s] | -2.00 (0.43) | -2.31 (0.62) | -2.11 ± 0.54 | -2.07 (0.53) | -1.98 (0.46) |
|  | *SponB %Δ* | | 15.97 (19.38) | 6.57 (21.15) | 4.99 (24.68) | -0.36 (16.28) |
|  | PMaxMin [cm/s] | 4.02 (0.87) | 4.53 (1.13) | 4.20 (1.04) | 4.20 (1.02) | 3.95 (0.88) |
|  | *SponB %Δ* | | 13.43 (18.42) | 5.45 (20.03) | 6.32 (25.89) | -0.85 (15.67) |
| **c2CSF**  (0.6 ~1.7 Hz) | PMax  [cm/s] | 1.06 (0.26) | 1.16 (0.29) | 1.22 (0.36) | 1.21 (0.40) | 1.07 (0.26) |
|  | *SponB %Δ* | | 12.05 (25.59) | 16.68 (31.08) | 15.76 (31.79) | 3.16 (23.15) |
|  | PMin  [cm/s] | -1.05 (0.26) | -1.16 (0.30) | -1.23 (0.36) | -1.24 (0.42) | -1.08 (0.31) |
|  | *SponB %Δ* | | 13.28 (23.63) | 18.05 (28.91) | 19.73 (36.70) | 4.68 (25.75) |
|  | PMaxMin [cm/s] | 2.11 (0.51) | 2.32 (0.58) | 2.45 (0.72) | 2.45 (0.81) | 2.15 (0.57) |
|  | *SponB %Δ* | | 12.55 (24.07) | 17.25 (29.43) | 17.67 (34.00) | 3.83 (23.94) |

**Table S5.** Time domain peak maximum, peak minimum, and peak maximum to peak minimum metrics were calculated in addition to averaged peak CSF metrics (mean, SD, and %Δ compared to SponB). SponB: spontaneous breathing, SlowB: slow breathing, DAB: seep abdominal breathing, DDB: deep diaphragmatic breathing, DCB: deep chest breathing, rCSF: respiratory-CSF, c1CSF: 1st cardiac harmonic component of CSF, c2CSF: 2nd cardiac harmonic component of CSF, APMax: averaged peak maximum, APMin: averaged peak minimum, APMaxMin: averaged peak minimum to averaged peak minimum.
